# Recurrence risk prediction model for hepatitis B virus-associated hepatocellular carcinoma patients: a systematic review and meta-analysis

**DOI:** 10.3389/fonc.2026.1777061

**Published:** 2026-03-06

**Authors:** Ke-Hao Zhao, Jiajun Liu, Yun-Shan Chen, Wen-Ting Yi, Juan-Juan Liu, Ying Zeng

**Affiliations:** 1 Department of International and Humanistic Nursing, Hunan Science Popularization Education Base, School of Nursing, Hengyang Medical School, University of South China, Hengyang, China; 2 School of Rehabilitation Medicine and Health, Hunan University of Medicine, Huaihua, China; 3 Hunan Engineering Research Center for Early Diagnosis and Treatment of Liver Cancer, Cancer Research Institute, Hunan Province Key Laboratory of Tumor Cellular & Molecular Pathology, Cancer Research Institute; Hengyang Medical School, University of South China, Hengyang, China

**Keywords:** hepatitis B virus-related hepatocellular carcinoma, meta-analysis, recurrence, risk prediction model, systematic review

## Abstract

**Background:**

Hepatitis B virus-associated hepatocellular carcinoma (HBV-HCC) is characterized by high postoperative recurrence rates. Although numerous recurrence prediction models exist, their performance and clinical utility remain uncertain.

**Objective:**

To systematically evaluate the performance and methodological quality of existing recurrence risk prediction models for HBV-HCC patients.

**Methods:**

We searched PubMed, Web of Science, Embase, Scopus, and OVID databases. Data were extracted following the CHARMS checklist, and the PROBAST tool was used to assess the risk of bias. A meta-analysis of the C-index from validation cohorts was performed using a random-effects model.

**Results:**

A total of 22 studies, encompassing 22 models, were included. Regarding the modeling methodology, 20 models were developed using the Cox proportional hazards regression model, one used a logistic regression model, and one utilized machine learning (ML). All 22 studies exhibited a high risk of bias, predominantly originating from the analysis domain. The meta-analysis revealed a pooled C-index of 0.73 (95% CI: 0.70-0.75) in the validation cohorts. The most frequently used predictors were MVI, AFP, tumor size, tumor number, and HBV-DNA.

**Conclusion:**

Existing recurrence prediction models for HBV-HCC demonstrate moderate predictive accuracy but are universally affected by a high risk of bias. This limits their reliability and applicability in current clinical practice. Future research should emphasize methodological rigor and conduct multicenter external validation before applying models in clinical practice.

**Systematic Review Registration:**

https://www.crd.york.ac.uk/PROSPERO/, identifier CRD42025629973.

## 1 Introduction

Primary liver cancer is a global health threat, ranking as the sixth most common and third most deadly malignancy worldwide (1). Hepatocellular carcinoma (HCC), as the predominant subtype, is particularly closely associated with hepatitis B virus (HBV) infection (2). Globally, especially within the Asia-Pacific region, HBV infection accounts for more than 50% of HCC cases (2, 3). In contrast to metabolic or alcohol-associated HCC, driven primarily by lipotoxicity and oxidative stress, HBV-associated HCC features a distinct immune microenvironment (4). The persistent viral infection creates a field effect of chronic inflammation and fibrosis, fostering an immunosuppressive niche enriched with regulatory T cells and exhausted cytotoxic T lymphocytes (4). This unique biological context involves viral DNA integration and continuous hepatic necroinflammation, which fundamentally differentiates the recurrence biology of HBV-HCC from that of non-viral etiologies (5). Despite continuous advancements in curative therapies such as hepatic resection, liver transplantation, and local ablation, the prognosis for patients with hepatitis B virus-associated hepatocellular carcinoma (HBV-HCC) remains poor (6). The primary reason is the high postoperative recurrence rate, which ranges from 50 to 70% within five years (7, 8). Notably, the biological mechanism of recurrence in HBV-related HCC is distinct from other etiologies. Recurrence typically manifests in two forms: intrahepatic metastasis (IM) derived from the primary tumor and multicentric carcinogenesis (MC) arising from the background of chronic hepatitis and cirrhosis. This dual mechanism implies that early recurrence is predominantly tumor-derived, whereas late recurrence is often driven by the pro-carcinogenic ‘‘field effect’’ of the liver background (9–11). Consequently, accurately predicting recurrence risk to guide individualized management has become a major challenge in current clinical practice. To address this need, many prediction models have been developed. These models aim to quantify the probability of postoperative recurrence for individual patients by integrating various factors, including clinical characteristics, pathological indicators, and imaging features (12–14). However, only prediction models that have undergone comprehensive validation can effectively assist clinicians in identifying high-risk individuals, facilitating more precise treatment and follow-up, ultimately reducing healthcare expenditures (15–17). The methodological quality and clinical applicability of these models remain unclear. Therefore, this study aimed to systematically evaluate the performance and methodological rigor of existing recurrence risk prediction models for HBV-HCC patients.

## 2 Methods

The study protocol was registered in PROSPERO (University of York), with the registration number CRD42025629973.

### 2.1 Search strategy

The study strictly adhered to Preferred Reporting Items for Systematic Reviews and Meta-Analyses (PRISMA) 2020 statement (18). To ensure the precision and comprehensiveness of the search, we utilized the PICOTS framework (19) to define the research question and construct the search strategy. The specific components were as follows:

P (population): HBV-HCC patients.

I (index model): All available prediction models.

C (comparator model): Not applicable.

O (outcome): Postoperative recurrence.

T (timing): Following anticancer therapy.

S (Setting): Inpatient or outpatient settings.

We searched five databases, including PubMed, Web of Science, Embase, Scopus, and OVID, from the inception to September 12, 2025. The search strategy combined subject headings and free-text words, focusing on two primary concepts: ‘hepatitis B virus-associated hepatocellular carcinoma’ and ‘prognosis.’ Additionally, a citation search was conducted on Google Scholar.

### 2.2 Inclusion and Exclusion Criteria

Inclusion criteria:

(1) patients aged ≥18 years with a pathological diagnosis of HBV-HCC; (2) studies focusing on clinical prediction models; (3) models that the predicted outcome was HCC recurrence; (4) models that included at least two predictors; (5) studies that developed or updated and validated a prediction model; (6) internal validation was defined as performance assessment within the same source population, including random split-sample, cross-validation, or bootstrapping; (7) external validation was defined as testing the model in a plausibly related but distinct cohorts, including geographically or temporal validation.

Exclusion criteria:

(1) full text unavailable; (2) prediction models developed based on systematic reviews; (3) incomplete data or inability to extract key metrics; (4) non-English language publications; (5) reviews, letters, conference abstracts, books, or expert opinions; (6) studies that only screened for risk factors for recurrence without developing a model; (7) diagnostic models; and (8) models that were not validated.

### 2.3 Study selection

After the systematic database search, duplicate records were removed using EndNote 21.0 software (Clarivate). Two independent reviewers screened the titles and abstracts of all initially retrieved articles. Subsequently, the full texts of potentially eligible articles were retrieved and assessed against the inclusion and exclusion criteria to determine final eligibility. Any disagreements between the two reviewers at any stage of the screening process were resolved through discussion to reach a consensus. If a consensus could not be reached, a third reviewer arbitrated the decision. Additionally, the citation search of all included studies was conducted to identify other potentially eligible studies.

### 2.4 Data extraction

Two independent researchers extracted the characteristics of the included studies and models by following the Checklist for Critical Appraisal and Data Extraction for Systematic Reviews of Prediction Modelling Studies (CHARMS) (20). The first part focused on the detailed characteristics of the included studies, including first author, year of publication, study design, enrollment periods, study settings, study region, and inclusion and exclusion criteria. The second part involved extracting the specifics of the prediction models, which included the modeling method, sample size of the development and validation cohorts, number of events, number of candidate and final predictors, methods for predictor selection, the final predictors, the prediction outcome, handling of missing data, model validation, model presentation, and the methods used to assess model performance.

### 2.5 Quality assessment

Two independent researchers systematically assessed the risk of bias (RoB) and applicability of all included studies using the Prediction Model Risk of Bias Assessment Tool (PROBAST) (21).PROBAST is the recommended tool for rigorously evaluating the methodological quality of studies on the development, validation, or updating of prediction models. Before the assessment, both researchers jointly studied and discussed the PROBAST checklist criteria. Any discrepancies that arose during the evaluation were resolved first through discussion; if a consensus could not be reached, the matter was settled through discussion with a third researcher.

The PROBAST tool comprises 20 signaling questions organized across four key domains: Participants, Predictors, Outcome, and Analysis. Each signaling question was answered as ‘‘Yes’’, ‘‘Probably Yes’’, ‘‘No’’, ‘‘Probably No’’, or ‘‘No Information’’, based on the information reported in the study. Following the PROBAST guidelines, the risk of bias for each domain was judged as ‘‘Low’’, ‘‘High’’, or ‘‘Unclear’’. A domain was rated as ‘‘Low risk’’ if all signaling questions were answered ‘‘Yes’’ or ‘‘Probably Yes’’; conversely, it was rated as ‘‘High risk’’ if any signaling question was answered ‘‘No’’ or ‘‘Probably No’’. A study’s overall risk of bias was judged to be ‘‘Low’’ only when all four domains were rated as ‘‘Low risk’’. Similarly, the applicability of each study was assessed across these four domains and rated as ‘‘Low concern’’, ‘‘High concern’’, or ‘‘Unclear concern’’.

### 2.6 Data analysis

To synthesize the predictive performance of the models, we conducted a meta-analysis of eligible studies using Stata MP 18.0 software to pool Harrell’s Concordance Index (C-index) from the validation cohorts. Given the anticipated methodological and clinical heterogeneity across studies, we used a random-effects model and the restricted maximum likelihood method to calculate the pooled C-index, along with the 95% Confidence Interval (CI). The heterogeneity was evaluated using the Cochrane’s Q test and the I² statistic (22). Meta-regression and subgroup analyses were used to assess the sources of heterogeneity. Sensitivity analysis was performed using the leave-one-out method to describe differences between meta-analytic summary C-indexes. Publication bias was assessed by examining the symmetry of the funnel plot and by performing Egger’s regression test, with a p-value < 0.1 considered indicative of significant bias (23).

## 3 Results

### 3.1 Literature search

**Figure 1:**
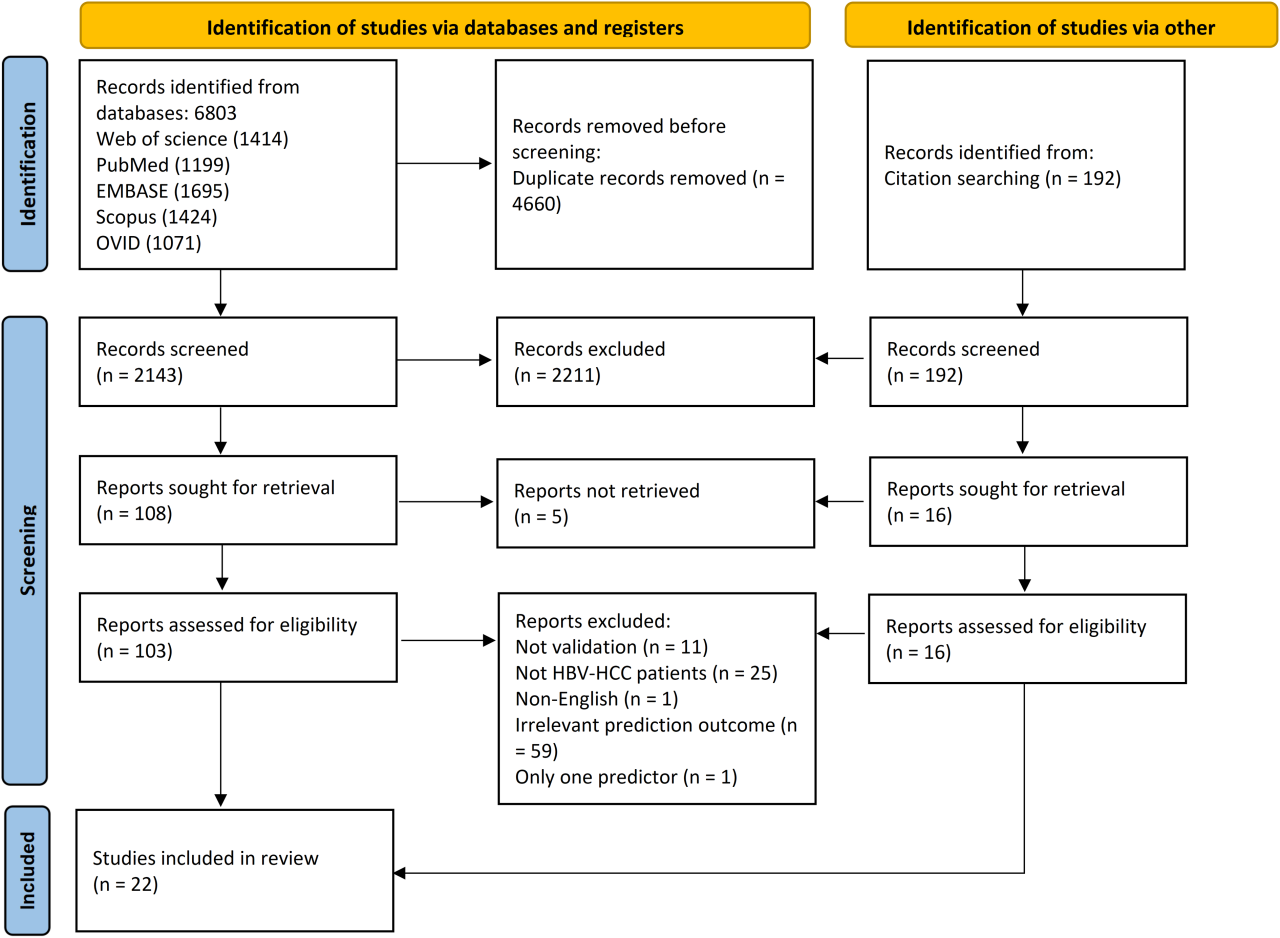
PRISMA flow diagram. Source: Page MJ, et al. (18).

Two independent researchers retrieved a total of 6,803 articles from five databases: Web of Science (n = 1,414), PubMed (n = 1,199), Embase (n = 1,695), Scopus (n = 1,424), and OVID (n = 1,071). All records were imported into EndNote 21.0 software (Clarivate), and 4,660 duplicates were removed. Based on the inclusion criteria, 2,035 articles were excluded after screening their titles and abstracts, leaving 103 for full-text review. An additional 192 articles were identified through citation searching in Google Scholar; after assessment, 16 of these were selected for full-text review. Ultimately, 22 studies were included in this review. A detailed flowchart of the literature screening process is presented in **Figure 1**. 

### 3.2 Characteristics of the studies

This systematic review ultimately included 22 studies, with publication years ranging from 2016 to 2025. Geographically, Asia was the primary focus of the studies, with 21 originating from China and one from South Korea. In terms of study design, the vast majority were retrospective studies, including 10 single-center and 10 multi-center studies. Additionally, 1 study used a professional database, and 1 study combined prospective and retrospective cohorts. For the 10 multi-center studies, the data sources were two independent hospitals (n = 6), three independent hospitals (n = 3), and four independent medical centers (n = 1). The detailed characteristics of all included studies are summarized in **Table 1** and **Supplementary Table 4**.

**Table 1:** Characteristics of the included studies.

Author, year	Study design	Enrolment period	Study setting	Study region
Ying Zhang 2025 (13)	Retrospective study	January 1, 2017, to December 30, 2020	Qilu Hospital of Shandong University, Shandong Provincial Hospital	China
Yu Zhu 2024 (14)	Retrospective study	January 1, 2012, to August 31, 2018	Inpatient information management system of the First Affiliated Hospital of University of Science, Technology of China, Taizhou Hospital affiliated with Wenzhou Medical University and Enze Hospital of Taizhou.	China
Yiqi Xiong 2024 (1) (24)	Retrospective study	January 2014 to December 2021	Beijing Youan Hospital	China
Yiqi Xiong 2024 (2) (25)	Retrospective study	January 2014 to December 2021	Beijing Youan Hospital and Beijing Ditan Hospital	China
Qi Wang 2024 (26)	Retrospective study	January 2014 to January 2020	Beijing You’an Hospital, Capital Medical University	China
Chongming Zheng 2023 (27)	Prospective study	March 2019 to December 2021	The First Affiliated Hospital of Wenzhou Medical University	China
Shilei Bai 2023 (28)	Retrospective study	January 2014 to December 2017	Shanghai Eastern Hepatobiliary Surgery Hospital, Jinling Hospital of Nanjing Medical University	China
Zehao Zheng 2023 (29)	Retrospective study	1 January 2014 to 1 January 2017	Guangdong Provincial People’s Hospital, Sun Yat-sen University Cancer Centre	China
Jiasi Zhang 2023 (30)	Retrospective study	2008 to 2011	Eastern Hepatobiliary Surgery Hospital (EHBH) the Second Military Medical University of China	China
Chao Wang 2022 (31)	Retrospective study	January 2015 to January 2020,	Affiliated Hospital from Qingdao University, Shulan Hospital	China
Zili Hu 2022 (32)	Retrospective study	April 2008 to September 2019	Sun Yat-sen University Cancer Center, The First Affiliated Hospital of Sun Yat-sen University, Hunan Provincial People’s Hospital, Shunde Hospital of Southern Medical University	China
Jin Gu 2022 (33)	Retrospective study	January 2008 to December 2014	Tongji Hospital	China
Wei Shuyao 2021 (34)	Retrospective study	January 2010 to May 2020	The 940th Hospital of Joint Logistics Support force of Chinese People’s Liberation Army, The First Affiliated Hospital of Medical School of Zhejiang University	China
Yujing Xin 2021 (35)	Retrospective study	March 2012 to December 2020	National Cancer Center, First Hospital of Shanxi Medical University	China
Mingyang Bao 2021 (36)	Retrospective study	July 1, 2014 to July 1, 2018	The First Affiliated Hospital of Zhejiang University School of Medicine, the 940th Hospital of Joint Logistics Support Force of Chinese People’s Liberation Army, The First Affiliated Hospital of Xinjiang Medical University, Urumqi	China
Xiangkun Wang 2019 (37)	Database analysis	Not mentioned	GSE14520	China
Jong Man Kim 2019 (38)	Retrospective study	April 2007 to September 2014	Samsung Medical Center	South Korea
Lingling He 2019 (39)	Retrospective study	(1) January 2012 and December 2014 (2) January 2015 and June 2015	Beijing Ditan Hospital, Capital Medical University	China
Abdulahad Abdulrab Mohammed Al-Ameri 2019 (40)	Database analysis	January 2015 to February 2019	China Liver Transplant Registry	China
Wei Qin 2018 (41)	Retrospective study	January 2005 to July 2017	The Third Affiliated Hospital of Sun Yat-sen University, The Affiliated Hospital/Clinical Medical College of Chengdu University, Huashan Hospital of Fudan University	China
Rui Liao 2018 (42)	Retrospective study	(1) January 2009 to December 2011 (2) 2008 and July 2009	The First and Second Affiliated Hospital of Chongqing Medical University	China
Ivan Fan-Ngai Hung 2016 (43)	Prospective and retrospective study	(1) June 2004 and December 2006 (2) July 2009 and June 2011	The University of Hong Kong, Queen Mary Hospital	China

### 3.3 Basic characteristics of the models

The 22 studies included in the review ultimately developed and validated a total of 22 independent prediction models. Twenty-one of these models were based on classical statistical methods, including the Cox proportional hazards regression model (n = 20) and the logistic regression model (n = 1). Only 1 study utilized machine learning (ML) to construct a model. With 9 models predicting recurrence rate, the outcome for the remaining 13 models was recurrence-free survival (RFS). For subsequent analyses, models, rather than studies, were treated as the independent units for data synthesis and statistical analysis. The detailed characteristics of each model are provided in the **Supplementary Table 5**.

### 3.4 Modeling predictor selection

**Figure 2:**
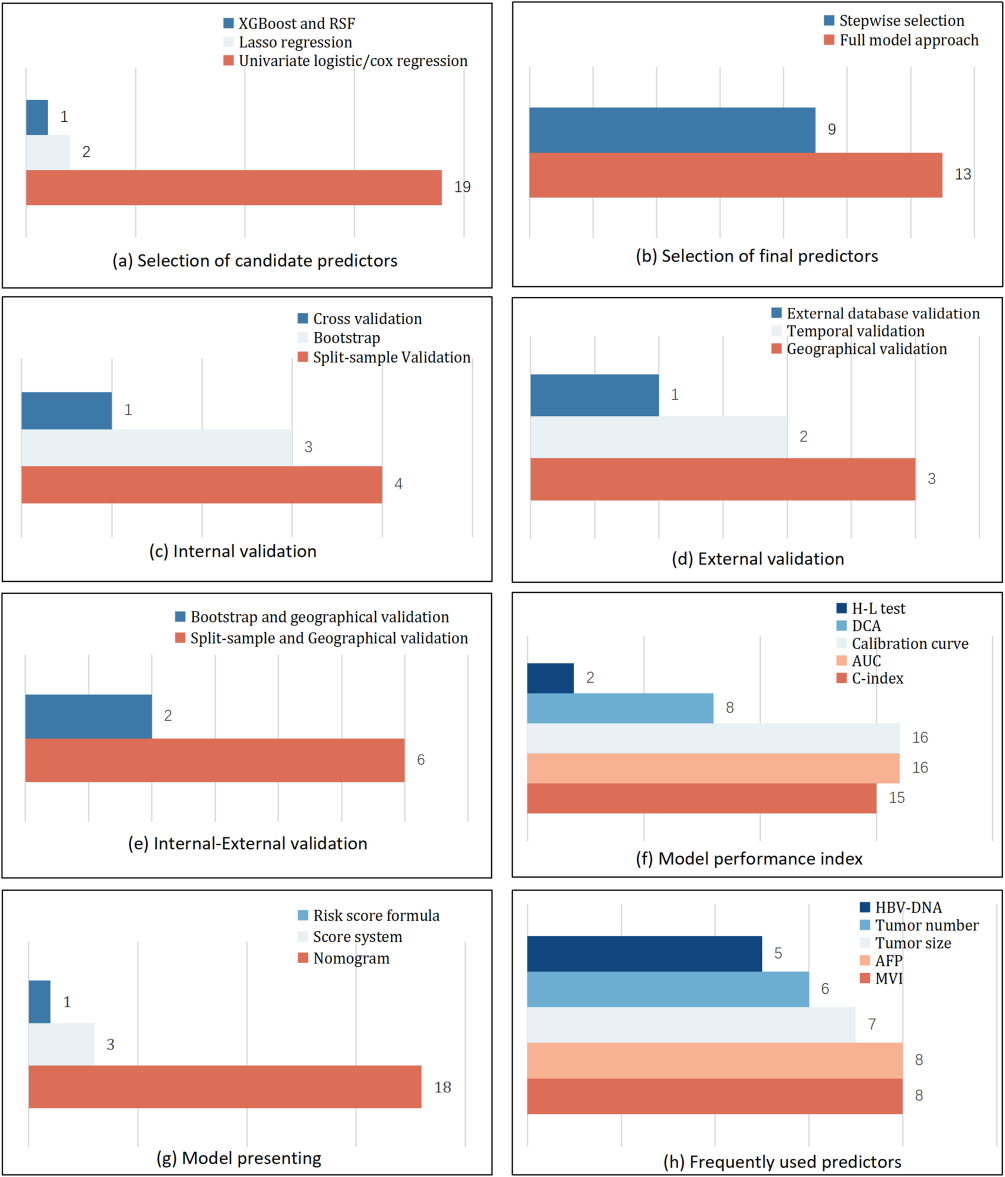
Basic information of included models.

Regarding the methods for selecting candidate predictors, the majority of models (n = 19) employed classic univariate analysis (univariate logistic or Cox regression). 2 models used Lasso regression, and 1 model utilized Extreme Gradient Boosting (XGBoost) and Random Survival Forest (RSF). For the selection of final predictors, 13 models adopted a full model approach, while the remaining 9 models used a stepwise selection method (**Figures 2a, b**).

### 3.5 Model validation

In terms of model validation, all 22 prediction models were validated using strategies that can be categorized into three types. Eight models (8/22) were validated internally using methods such as split-sample validation (n = 4), bootstrapping (n = 3), and cross-validation (n = 1). Six models (6/22) underwent external validation, which included geographical validation (n = 3), temporal validation (n = 2), and external database validation (n = 1). Furthermore, the remaining 8 models combined both internal and external validation, specifically split-sample with geographical validation (n = 6) and bootstrapping with geographical validation (n = 2) (**Figures 2c–e**).

### 3.6 Model performance index and presence

The models were evaluated using multi-dimensional performance metrics. Discrimination was primarily assessed by the C-index (n = 15) and the AUC (n = 16). For calibration, 16 models were visually assessed using calibration curves, and 2 models applied the Hosmer-Lemeshow (H-L) test. Regarding clinical usefulness, 8 studies performed Decision Curve Analysis (DCA) (**Figure 2f**).

Regarding model presentation, the nomogram was the most common format (18/22). Notably, one study further developed its nomogram into an online calculator to enhance clinical accessibility. Other presentation formats included a risk score formula (1/22) and scoring systems (3/22) (**Figure 2g**).

### 3.7 Predictors and sample size

All models used a total of 78 different predictors. The most frequently occurring predictors were microvascular invasion (MVI) (n = 8), alpha-fetoprotein (AFP) (n = 8), tumor size (n = 7), tumor number (n = 6), HBV-DNA level (n = 5), aspartate aminotransferase (n = 4), and the Barcelona Clinic Liver Cancer (BCLC) stage (n = 3) (**Figure 2h**).

**Figure 3:**
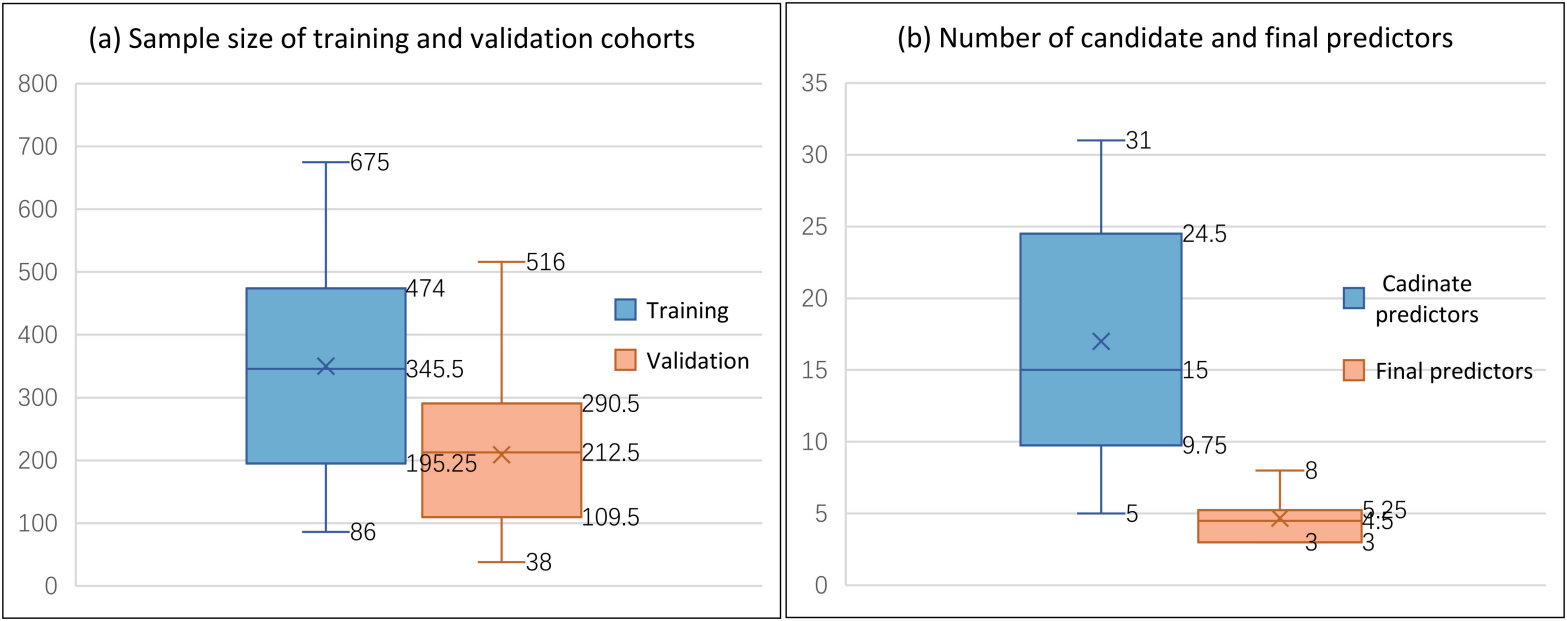
Model sample size and the number of predictors.

The 22 prediction models included in this review involved a total of 11,534 HBV-HCC patients. Regarding sample size reporting, 4 models only presented the overall sample size without a clear demarcation between development and validation cohorts. For the remaining 18 models that did report these numbers distinctly, the median sample sizes (min, max) for the development and validation cohorts were 345.5 (86, 675) and 212.5 (38, 516), respectively. In terms of the number of predictors, the median number (min, max) of candidate predictors was 15 (5, 31), while the median number (min, max) of final predictors included in the models was 4.5 (3, 8) (**Figure 3**).

### 3.8 Literature quality assessment

**Figure 4:**
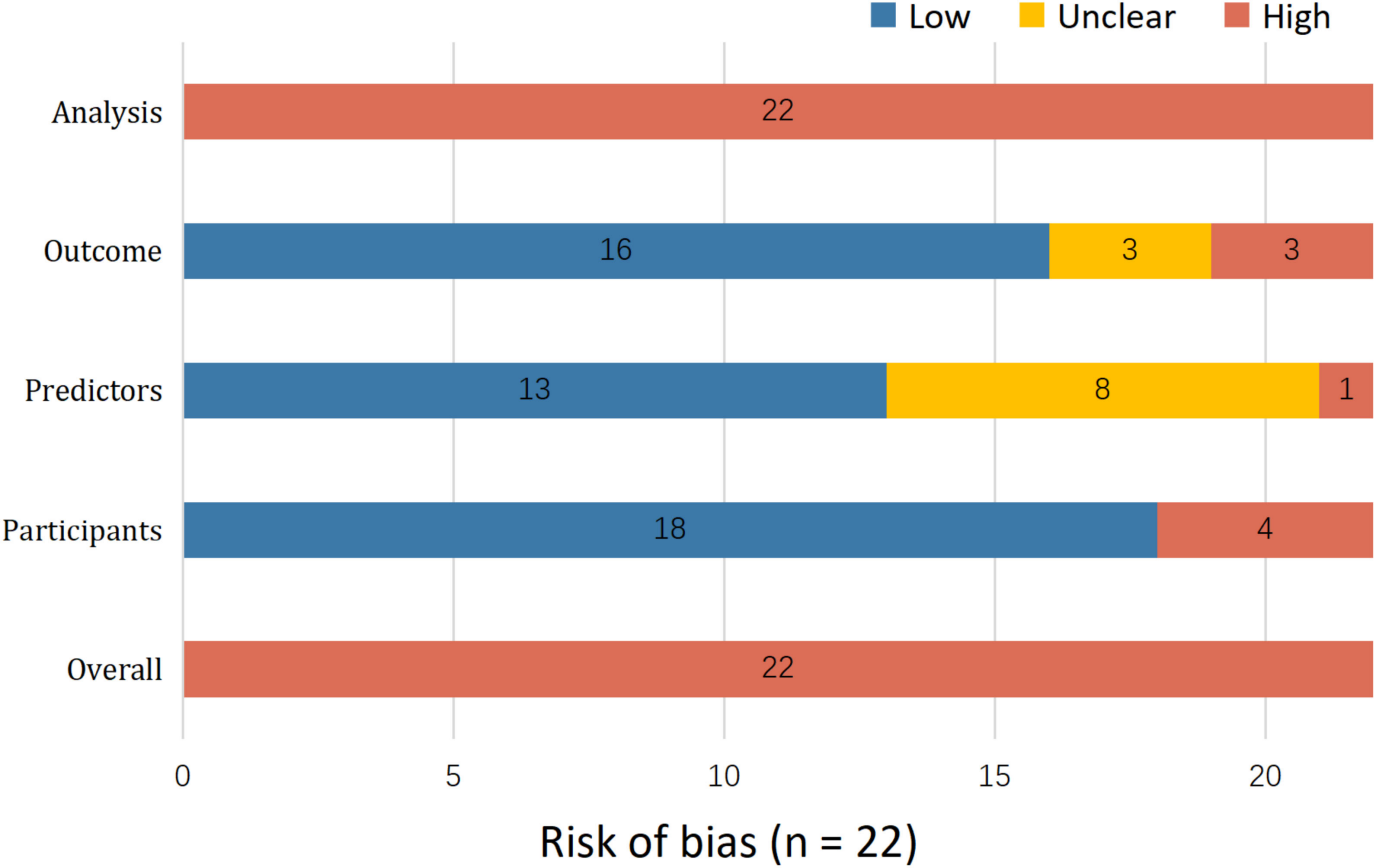
Risk of bias assessment.

Quality assessment revealed a universal high risk of bias, systematically stemming from the analysis domain. Major sources of bias included the use of complete-case analysis for missing data (n = 17) and univariate screening for predictor selection (n = 19), both of which compromise model stability. Furthermore, the inappropriate categorization of continuous variables (n = 12) and the frequent omission of calibration assessments (n = 6) further undermined methodological rigor. Selection bias was also identified in two studies due to subgroup exclusions. Consequently, the predictive accuracy of these models is likely overestimated, limiting their reliability for current clinical practice (**Figure 4**, **Supplementary Figure 6**).

### 3.9 Data analysis

**Figure 5:**
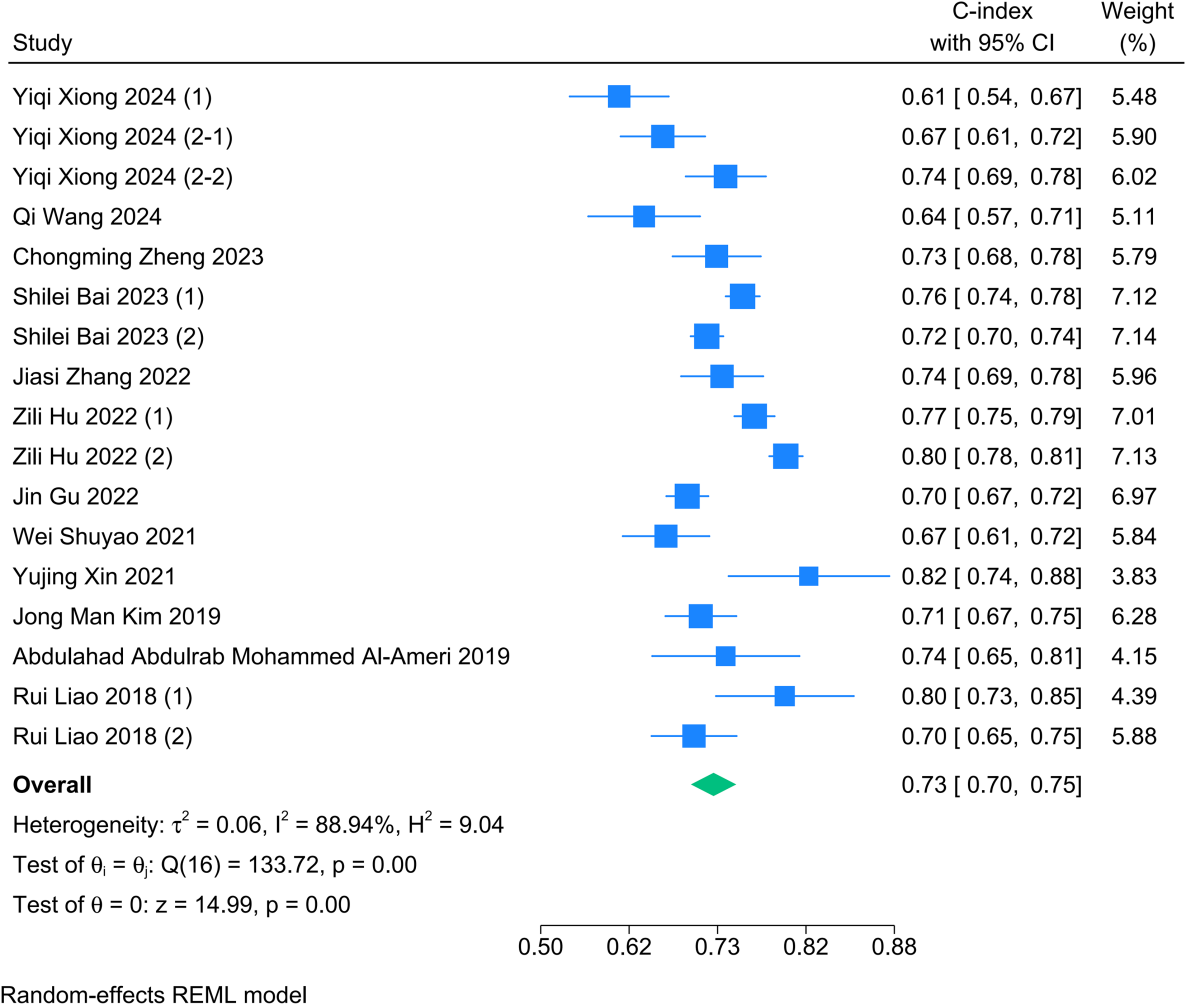
Meta-analysis of C-index.

A meta-analysis was conducted on the discrimination performance of the prediction models as reported in their validation cohorts. The C-index values reported by the models ranged from 0.609 to 0.820. The pooled C-index value, calculated using a random-effects model, was 0.73 (95% CI: 0.70-0.75), indicating a moderate level of discrimination (**Figure 5**). The heterogeneity test result was I² = 88.94%. A subgroup analysis revealed that the prediction outcome was a source of heterogeneity (*P* = 0.022). Further meta-regression indicated that sample size (β = 0.00036, 95% CI: 0.00012 to 0.00059, *P* = 0.003, *R^2^* = 49.03%) was a significant source of heterogeneity. The sensitivity analysis revealed that the total effect value was robust. To assess potential publication bias, we employed a combination of Egger’s test and funnel plots. The results detected no statistically significant publication bias or small-study effects for the C-index (*P* = 0.8611). The symmetrical funnel plots further support this conclusion.

## 4 Discussion

This study systematically evaluated published recurrence risk prediction models for HBV-HCC patients. The included studies were published between 2016 and 2025, indicating that recurrence risk prediction is receiving growing attention. The majority of these studies were conducted in China, which likely reflects the country having the highest number of HCC patients globally (44). The majority of models (21/22) utilized classical regression methods, likely due to their relative interpretability and ease of implementation (45). While emerging evidence suggests machine learning may offer superior accuracy, its advantage is contingent upon large datasets and robust feature engineering (46). Crucially, algorithmic complexity cannot compensate for methodological flaws; rigorous study design remains the cornerstone of model reliability regardless of the statistical approach used.

The meta-analysis indicated a pooled C-index of 0.73 (95% CI: 0.70-0.75) across the included validation cohorts, reflecting a modest level of discrimination overall. Sensitivity analysis confirmed the stability of the pooled effect size, indicating that no single study significantly impacted the overall findings. Notably, substantial heterogeneity was observed (I² = 88.94%, *P* < 0.001), reflecting considerable variations in the effect estimates across the studies. Therefore, we conducted a subgroup analysis and meta-regression to investigate the origin of significant heterogeneity, finding that the prediction outcome (*P* = 0.022) and sample size (*P* = 0.003) were critical factors influencing heterogeneity. Furthermore, the observed variation in C-indices ranging from 0.61 to 0.82 should be interpreted with caution. This heterogeneity may not solely reflect the superiority of specific modeling strategies. Instead, it likely reflects differences in case mix across the validation cohorts. Performance metrics like the C-index are sensitive to the spectrum of disease severity in the validation population. In cohorts with a broad range of disease stages, it is inherently easier for a model to distinguish between patients who will recur and those who will not, resulting in artificially higher C-index values. Conversely, in more homogeneous cohorts, discrimination is more challenging, often yielding lower performance metrics regardless of the model’s intrinsic quality. Therefore, a lower C-index in some studies may reflect a harder-to-predict population rather than a flawed model.

Our PROBAST assessment revealed a stark contrast: while the participant domain often showed low risk of bias, the analysis domain was universally high risk. This discrepancy underscores a critical insight: the primary bottleneck in current oncology predictive research is not the scarcity of high-quality clinical cohorts, but rather a limitation in statistical literacy and methodological rigor. Consequently, valuable clinical data is often compromised by suboptimal analytical handling, such as the inappropriate handling of missing data and continuous variables. Future progress, therefore, depends more on fostering closer collaboration between clinicians and methodologists to ensure appropriate statistical handling.

Model performance is intrinsically linked to the rigor of the development methodology. Therefore, a detailed and careful description of the research methodology is necessary to facilitate better dissemination.

By integrating the risk of bias factors from modeling studies, our critical appraisal identified the following systemic issues:

A significant proportion of multi-center studies did not clearly specify the methods and standards for the measurement and evaluation of predictors across different centers. This makes it difficult to standardize and compare the reliability of results from different settings. Strictly standardizing and defining the evaluation criteria for predictors is crucial.

Most studies (17/22) handled missing data using complete case analysis, a simple and common approach. However, for prediction models with RFS outcomes, complete case analysis substantially reduces sample size. This approach leads to significant information loss during model development (45). Building a prediction model on such an artificially selected sub-sample set is highly likely to introduce bias into the results (47). For handling missing data, researchers should consider using imputation method (45, 47).

Regarding statistical rigor and reporting quality, we have summarized the following issues:

Insufficient sample size: Only four models (28, 30, 33, 34) met the robust criteria of having an Events Per Variable (EPV) ratio greater than 20 (21, 48, 49) and a validation set sample size of over 100 (50–52). Insufficient sample size, particularly when the number of outcome events is too low relative to the number of predictors, is a fundamental cause of model overfitting and overly optimistic performance estimates (21).

Outdated predictor selection methods: Before incorporating candidate predictors into multivariate analysis modeling, the majority of studies (19/22) still relied on univariate analysis for predictor selection. When the number of events is small relative to the number of predictors, standard regression may produce overfitted models with inaccurate predictions. Using penalized regression can improve the accuracy of risk prediction (53).

Insufficient and non-standardized validation strategies: Although all models reported a validation process, the rigor and quality of these validations were inconsistent. Research by Steyerberg et al. (54) points out that prediction models should undergo internal-external validation; the purpose of internal validation is to quantify the degree of optimism in model performance within a single population, and many external validation failures can be preempted by rigorous internal validation (54, 55). However, 10 studies employed split-sample validation. This is not a reliable method for internal validation, and bootstrapping should be the preferred approach (54). The purpose of external validation is to assess the generalizability of a model to similar or related populations. When a prediction model is developed for application to new individuals, its value depends on its performance beyond the development sample sets (55–57). While 14 studies claimed to have performed external validation, most were cross-temporal or cross-institutional validations within the same geographical region, merely testing for reproducibility (54). Truly independent external validations based on heterogeneous populations remain scarce, leaving the model’s generalizability unreliably assessed (58).

Incomplete reporting of model performance metrics: Evaluating a risk prediction model requires a comprehensive set of metrics. The assessment of discrimination and calibration should be considered basic requirements. If a model is intended to support clinical decisions, metrics from decision analysis must also be reported (59). Ultimately, the goal of developing recurrence risk prediction models for HBV-HCC patients is to serve clinical decision-making. However, the included studies predominantly focused on reporting model discrimination, using the C-index (n = 15) or AUC (n = 16). Yet, nearly one-third (6/22) of the studies completely omitted an assessment of model calibration. If a model’s predicted probabilities deviate significantly from actual observed outcomes, its clinical application is not only unhelpful but could even be misleading (60). While discrimination separates patients with and without the outcome, calibration ensures that the predicted probabilities accurately reflect the observed risks. This distinction is clinically vital because a model with good discrimination but poor calibration can be dangerous in practice. Specifically, the calibration slope reflects the spread of the estimated risks. A slope < 1 suggests that the model yields predictions that are too extreme, causing low-risk patients to be perceived as lower risk and high-risk patients as higher risk than they actually are. Meanwhile, the calibration intercept assesses whether the risk predictions are systematically too high or too low on average. Clinically, poor calibration is dangerous. Systematic overestimation could lead to overtreatment, while underestimation might result in insufficient surveillance for patients who actually have a high recurrence risk (50, 61, 62). Similarly, Decision Curve Analysis (DCA), which assesses the clinical utility and net benefit of a model (63), was reported in only a few studies (n = 8), reflecting the one-sided nature of current model evaluation practices.

A qualitative synthesis of these studies reveals that the potential clinical impact of these models primarily focuses on four domains:

Adjuvant Therapy Decision-making: Zhang et al. (30) proposed that identifying patients at high risk of early recurrence is essential for initiating aggressive adjuvant therapies to mitigate poor outcomes. Furthermore, Wang et al. (31) extended this precision approach to liver transplantation recipients; by integrating coagulation biomarkers (fibrinogen and D-dimer), their model not only predicts recurrence but also implies a potential therapeutic window for strict anticoagulation management or targeted adjuvant therapy in high-risk individuals. Collectively, these studies illustrate how prognostic models can serve as decision-aids to tailor therapeutic intensity according to individual risk profiles.

Dynamic Surveillance Strategies: Beyond static follow-up schedules, current research advocates for risk-stratified and dynamic surveillance strategies to optimize healthcare resource allocation. Zhang et al. (13) emphasized the clinical necessity of intensive monitoring for patients identified as high-risk for early recurrence.

Etiological and Immunological Management: Xiong et al. (25) demonstrated that elevated quantitative HBsAg levels serve as a robust prognostic factor. Biologically, high antigen load often correlates with immune exhaustion and impaired antigen presentation efficiency. Consequently, their model identifies a specific subgroup of patients with high antigen burden who may require intensified antiviral strategies or immunotherapies to reverse immune tolerance and reboot anti-tumor surveillance. Complementarily, Hu et al. (32) utilized systemic inflammatory biomarkers to construct a risk stratification tool. Their findings underscore that for patients exhibiting a high inflammatory index, clinical management should extend beyond resection to include immunomodulation or anti-inflammatory interventions.

Perioperative Risk Management: Bao et al. (36) extended the scope of prediction beyond tumor recurrence to immediate surgical outcomes. Their study demonstrated that identifying patients at high risk for postoperative complications via a nomogram could guide the implementation of preventive nursing and intensified perioperative monitoring. By optimizing the short-term recovery trajectory and reducing surgical morbidity through targeted interventions, this approach aims to secure the physiologic reserve required for long-term survival.

Despite these diverse proposals, a critical gap remains. Our assessment indicates that none of the reviewed models provided evidence of actual implementation in clinical workflows. The net benefit reported remains theoretical. Future research must bridge this gap by conducting impact studies to evaluate whether model-assisted decision-making truly alters physician behavior and improves patient outcomes compared to standard care.

Regarding predictors, the 22 models included in this review incorporated 78 distinct predictors. Among these, MVI, tumor size, tumor number, AFP level, and HBV-DNA load were the five most frequently used variables. To elucidate their biological relevance, we categorized the most frequently identified predictors into three biological domains: tumor biology, liver disease activity, and host immunity.

Predictors related to tumor biology predominantly reflect tumor aggressiveness and the potential for occult metastasis, acting as primary drivers of early recurrence. MVI serves as a direct histological marker of invasive potential. A higher grade of MVI is strongly correlated with the presence of disseminated tumor cells in the portal circulation, which are the seeds of early intrahepatic metastasis (64). Elevated AFP levels indicate poorer tumor differentiation, increased proliferation, greater invasiveness, and a higher risk of recurrence. Similarly, a high tumor burden suggests active proliferation and a susceptibility to micrometastasis and multicentric origin, resulting in a significantly elevated risk of recurrence (65–67).

Liver disease activity reflects the “field effect” of the carcinogenic liver background, which is closely linked to late recurrence (68). HBV-DNA load and inflammatory markers signify active viral replication and chronic hepatic necroinflammation. High viral load creates a pro-inflammatory microenvironment, promoting *de novo* carcinogenesis while fostering an immunosuppressive niche via cytotoxic T lymphocyte exhaustion (69). This distinction explains why models heavily weighted with tumor factors may exhibit reduced predictive accuracy for late recurrence, as they fail to capture the *de novo* carcinogenic risk driven by the underlying liver disease.

Host immunity represents the dynamic interplay between immune surveillance and tumor evasion. While currently few models (25, 29, 32, 42) explicitly incorporate immune markers, the predictive value of systemic inflammatory indices and antigen load in some studies highlights the critical role of the immune microenvironment. Central to this is the liver’s unique antigen processing and presentation (APP) machinery—involving Kupffer cells and LSECs—which maintains the balance between immune tolerance and activation (70, 71). In HBV-HCC, chronic infection disrupts these pathways (e.g., via modulation of MHC class I/II expression or trogocytosis), creating an immune-evasive milieu that facilitates the survival of micrometastatic foci (72, 73). Since conventional morphological predictors fail to capture these complex biological dimensions, the moderate performance of current models is unsurprising. Therefore, integrating markers of antigen presentation activity to characterize the hepatic ‘‘soil’’ may overcome current prediction bottlenecks.

A critical distinction must be made between preoperative decision models and postoperative prognostic models based on the timing of predictor availability. As highlighted by our results, MVI is one of the most powerful predictors of recurrence; however, its reliance on postoperative pathology limits its utility in preoperative surgical planning. We identified four distinct preoperative decision models in the included studies. Al-Ameri et al. (40) constructed the ‘‘5–8 score’’ to refine candidate selection for liver transplantation (LT). Unlike the binary Milan criteria, their model dictates a stratified management strategy: low-risk patients (score 0-5) are cleared for direct LT; medium-risk patients (score 6-8) are recommended for pre-LT downstaging and postoperative mTOR inhibitor therapy; while high-risk patients (score >8) should be initially excluded from LT and prioritized for neoadjuvant therapy. Qin et al. (41) challenged the conventional contraindication for surgery in intermediate-stage cancer. Their DFT score (Differentiation, FIB-4, Tumor volume) identifies a specific subgroup of BCLC B/C patients with low scores who can still derive survival benefit from ‘aggressive hepatectomy,’ whereas high-score patients are advised to undergo TACE or hepatic arterial infusion to downstage the tumor first. Notably, they advocated for preoperative biopsy to obtain histological differentiation, thereby advancing this pathological predictor into the preoperative decision phase. Similarly, Gu et al. (33) focused on the ‘‘soil’’ of the liver. By integrating the non-invasive Cirrhotic Severity Scoring, their nomograms allow surgeons to preoperatively weigh the risk of tumor recurrence against the severity of background liver disease, assisting in the delicate balance between resection extent and hepatic reserve preservation. Future research should explicitly define the target clinical window to maximize clinical applicability.

We strongly recommend that future studies utilize large sample sizes, multi-center, prospective cohorts for model validation. This is essential for minimizing selection bias and enhancing the generalizability of the models. During the model development and validation phases, researchers should strictly adhere to the Transparent Reporting of a Multivariable Prediction Model for Individual Prognosis or Diagnosis (TRIPOD) statement (47). We suggest using the methods proposed by Richard D. Riley (74) for rigorous sample size estimation to ensure the model has adequate statistical power. In addition to performing internal-external validation, the consistency of predictor effects across different subsets can be directly assessed using statistical tests (54). This approach can provide deeper insights into a model’s generalizability than validation alone. Regarding model predictive performance, comprehensive and standardized evaluation metrics should be provided, including at least: discrimination, calibration, and clinical utility. Recurrence dynamics in HBV-HCC are not constant; they differ significantly between early and late phases. Static discrimination values, such as a single C-index, obscure this biological distinction. Consequently, we were unable to assess how well these models perform at specific high-risk windows. We strongly advocate moving beyond static baselines to adopt dynamic prediction approaches, such as reporting time-dependent AUCs or employing landmark analysis. This shift is essential to capture the time-varying risk profiles of patients and to guide stage-specific surveillance strategies. Finally, we advise clinicians to exercise caution when applying current recurrence risk prediction models for HBV-HCC patients. These models should be used as auxiliary references for clinical decision-making, rather than as the sole basis for decisions. When using a recurrence risk prediction model, it is crucial to understand the population upon which it was developed. There should be no significant differences in clinical characteristics between the population used to develop the prediction model and the population in which the model is being applied (15).

## 5 Conclusion

While current recurrence prediction models for HBV-HCC demonstrate acceptable discrimination (pooled C-index: 0.73), their clinical utility is severely compromised by widespread methodological flaws, particularly inappropriate predictor selection and handling of missing data. Most models likely suffer from overfitting and lack rigorous external validation. Given these limitations, current models should be regarded as exploratory tools rather than practice-changing instruments. Furthermore, the reliance on static tumor-burden markers fails to capture the dynamic risk of late recurrence driven by the hepatic immune microenvironment. Therefore, simply developing more retrospective models using standard regression is of diminishing value. Future research must prioritize methodological rigor, incorporate dynamic prediction tools, and integrate novel biomarkers to truly bridge the gap between statistical significance and clinical implementation.

## 6 Limitations

This study also has several limitations. First, the included studies had high statistical heterogeneity, and we only investigated two sources of heterogeneity, missing others. Second, our eligibility criteria required models to include at least two predictors. We acknowledge that this distinction is methodological rather than clinical. Single strong predictors can theoretically offer high prognostic value and simplicity. However, our review strictly focused on multivariable models to assess the synergistic performance of integrating multiple clinical variables, which represents a different level of complexity compared to single-factor studies. Third, most studies did not indicate the time-dependent area under the curve (t-AUC) values at specific time points in the model, we were unable to do further a subgroup analysis of the included models, limiting the meta-analysis’s direct explanatory ability. Fourth, the geographical origin of the included studies was predominantly concentrated in East Asia (21 from China and 1 from South Korea). It substantially restricts the external validity of these models. Consequently, their applicability to other high-prevalence regions, such as sub-Saharan Africa, or to Western populations remains uncertain due to potential differences in HBV genotypes, host genetic backgrounds, and healthcare systems. Therefore, caution is warranted when extrapolating these findings to non-Asian populations. Fifth, our search strategy was restricted to English-language literature. Since a significant proportion of HBV-HCC research originates from East Asia, where domestic language publications remain frequent, this exclusion criterion may have introduced a systematic bias. By limiting our review to English publications, we may have inadvertently filtered out local studies that represent different clinical settings or methodological approaches, thereby potentially affecting the representativeness of the real-world performance of these models in their native populations. Finally, although the statistical tests for publication bias were not significant, the existence of potential publication bias cannot be entirely ruled out, considering the generally small sample sizes of the included studies.

## Data Availability

The original contributions presented in the study are included in the article/**Supplementary Material**. Further inquiries can be directed to the corresponding authors.
