# Supplementary material for: Recurrence risk prediction model for hepatitis B virus-associated hepatocellular carcinoma patients: a systematic review and meta-analysis
Source: Front Oncol. 2026 Mar 6;16:1777061. doi: 10.3389/fonc.2026.1777061 (PMC13002444; doi:10.3389/fonc.2026.1777061)
Supplement: Supplementary file 1 [file DataSheet1.docx]

**Supplemental material**

Recurrence risk prediction model for Hepatitis B Virus-Related Hepatocellular Carcinoma Patients: A Systematic Review and Meta-Analysis

Table S1 PRISMA checklist

| **Section and Topic** | **Item #** | **Checklist item** | **Location where item is reported (section)** |  |  |
| --- | --- | --- | --- | --- | --- |
| **TITLE** | | |  |  |  |
| Title | 1 | Identify the report as a systematic review. | Pg 1 |  |  |
| **ABSTRACT** | | |  |  |  |
| Abstract | 2 | See the PRISMA 2020 for Abstracts checklist. | Pg 1 |  |  |
| **INTRODUCTION** | | |  |  |  |
| Rationale | 3 | Describe the rationale for the review in the context of existing knowledge. | Pg 3 |  |  |
| Objectives | 4 | Provide an explicit statement of the objective(s) or question(s) the review addresses. | Pg 3 |  |  |
| **METHODS** | | |  |  |  |
| Eligibility criteria | 5 | Specify the inclusion and exclusion criteria for the review and how studies were grouped for the syntheses. | Pg 4,5 |  |  |
| Information sources | 6 | Specify all databases, registers, websites, organisations, reference lists and other sources searched or consulted to identify studies. Specify the date when each source was last searched or consulted. | Pg 4 |  |  |
| Search strategy | 7 | Present the full search strategies for all databases, registers and websites, including any filters and limits used. | Pg 4 |  |  |
| Selection process | 8 | Specify the methods used to decide whether a study met the inclusion criteria of the review, including how many reviewers screened each record and each report retrieved, whether they worked independently, and if applicable, details of automation tools used in the process. | Pg 5 |  |  |
| Data collection process | 9 | Specify the methods used to collect data from reports, including how many reviewers collected data from each report, whether they worked independently, any processes for obtaining or confirming data from study investigators, and if applicable, details of automation tools used in the process. | Pg 5, 6 |  |  |
| Data items | 10a | List and define all outcomes for which data were sought. Specify whether all results that were compatible with each outcome domain in each study were sought (e.g. for all measures, time points, analyses), and if not, the methods used to decide which results to collect. | Supplement material Table S3, TableS4 and TableS5 |  |  |
|  | 10b | List and define all other variables for which data were sought (e.g. participant and intervention characteristics, funding sources). Describe any assumptions made about any missing or unclear information. | Supplement material Table S3, TableS4 and TableS5 |  |  |
| Study risk of bias assessment | 11 | Specify the methods used to assess risk of bias in the included studies, including details of the tool(s) used, how many reviewers assessed each study and whether they worked independently, and if applicable, details of automation tools used in the process. | Pg 6 |  |  |
| Effect measures | 12 | Specify for each outcome the effect measure(s) (e.g. Risk ratio, mean difference) used in the synthesis or presentation of results. | Pg 7 |  |  |
| Synthesis methods | 13a | Describe the processes used to decide which studies were eligible for each synthesis (e.g. tabulating the study intervention characteristics and comparing against the planned groups for each synthesis (item #5)). | Pg 6, 7 |  |  |
|  | 13b | Describe any methods required to prepare the data for presentation or synthesis, such as handling of missing summary statistics, or data conversions. | Pg 6, 7 |  |  |
|  | 13c | Describe any methods used to tabulate or visually display results of individual studies and syntheses. | Pg 6, 7 |  |  |
|  | 13d | Describe any methods used to synthesize results and provide a rationale for the choice(s). If meta-analysis was performed, describe the model(s), method(s) to identify the presence and extent of statistical heterogeneity, and software package(s) used. | Pg 6, 7 |  |  |
|  | 13e | Describe any methods used to explore possible causes of heterogeneity among study results (e.g. subgroup analysis, meta-regression). | Pg 6, 7 |  |  |
|  | 13f | Describe any sensitivity analyses conducted to assess robustness of the synthesized results. | Pg 6, 7 |  |  |
| Reporting bias assessment | 14 | Describe any methods used to assess risk of bias due to missing results in a synthesis (arising from reporting biases). | Pg 6, 7 |  |  |
| Certainty assessment | 15 | Describe any methods used to assess certainty (or confidence) in the body of evidence for an outcome. | Pg 6, 7 |  |  |
| **RESULTS** | | |  |  |  |
| Study selection | 16a | Describe the results of the search and selection process, from the number of records identified in the search to the number of studies included in the review, ideally using a flow diagram. | Pg 7 |  |  |
|  | 16b | Cite studies that might appear to meet the inclusion criteria, but which were excluded, and explain why they were excluded. | Figure 1 |  |  |
| Study characteristics | 17 | Cite each included study and present its characteristics. | Pg 8-13 and Table1 |  |  |
| Risk of bias in studies | 18 | Present assessments of risk of bias for each included study. | Pg 14, 15 |  |  |
| Results of individual studies | 19 | For all outcomes, present, for each study: (a) summary statistics for each group (where appropriate) and (b) an effect estimate and its precision (e.g. confidence/credible interval), ideally using structured tables or plots. | Pg 15, 16 |  |  |
| Results of syntheses | 20a | For each synthesis, briefly summarise the characteristics and risk of bias among contributing studies. | Pg 11-14 Figure 2 and Figure 3 |  |  |
|  | 20b | Present results of all statistical syntheses conducted. If meta-analysis was done, present for each the summary estimate and its precision (e.g. confidence/credible interval) and measures of statistical heterogeneity. If comparing groups, describe the direction of the effect. | Pg 15,16 |  |  |
|  | 20c | Present results of all investigations of possible causes of heterogeneity among study results. | Supplement material Figure S1 and Figure S2 |  |  |
|  | 20d | Present results of all sensitivity analyses conducted to assess the robustness of the synthesized results. | Supplement material Figure S3 |  |  |
| Reporting biases | 21 | Present assessments of risk of bias due to missing results (arising from reporting biases) for each synthesis assessed. | Supplement material Figure S4 and Figure S5 |  |  |
| Certainty of evidence | 22 | Present assessments of certainty (or confidence) in the body of evidence for each outcome assessed. | Pg 16 |  |  |
| **DISCUSSION** | | |  |  |  |
| Discussion | 23a | Provide a general interpretation of the results in the context of other evidence. | Pg 17, 18, 19, |  |  |
|  | 23b | Discuss any limitations of the evidence included in the review. | Pg 21 |  |  |
|  | 23c | Discuss any limitations of the review processes used. | Pg 21 |  |  |
|  | 23d | Discuss implications of the results for practice, policy, and future research. | Pg 20, 21 |  |  |
| **OTHER INFORMATION** | | |  |  |  |
| Registration and protocol | 24a | Provide registration information for the review, including register name and registration number, or state that the review was not registered. | Pg 3 |  |  |
|  | 24b | Indicate where the review protocol can be accessed, or state that a protocol was not prepared. | Pg 3 |  |  |
|  | 24c | Describe and explain any amendments to information provided at registration or in the protocol. | Pg 3 |  |  |
| Support | 25 | Describe sources of financial or non-financial support for the review, and the role of the funders or sponsors in the review. | Pg 22, 23 |  |  |
| Competing interests | 26 | Declare any competing interests of review authors. | Pg 22, 23 |  |  |
| Availability of data, code and other materials | 27 | Report which of the following are publicly available and where they can be found template data collection forms; data extracted from included studies; data used for all analyses; analytic code; any other materials used in the review. | Pg 22, 23 |  |  |

Table S2: Systematic Database Search strategy

| **Databases** | **Query** | **Results** |
| --- | --- | --- |
| PubMed (From database establishment to September 12, 2025) | (("Hepatitis B Virus-Related hepatocellular carcinoma"[Title/Abstract] OR "HBV-hepatocellular carcinoma"[Title/Abstract] OR "HBV-HCC"[Title/Abstract] OR "Hepatitis B virus-related HCC"[Title/Abstract] OR "HBV positive HCC"[Title/Abstract] OR "Hepatitis-B Virus-Associated Hepatocellular Carcinoma"[Title/Abstract] OR "HBV-Related Hepatocellular Carcinoma"[Title/Abstract] OR "HBV-associated hepatocellular carcinoma "[Title/Abstract])) AND (("Prognosis"[Title/Abstract] OR "prognostic factors"[Title/Abstract] OR "prognostic model*"[Title/Abstract] OR "Prognostic Risk Prediction"[Title/Abstract] OR "predict model"[Title/Abstract] OR "prediction model*"[Title/Abstract] OR "nomogram*"[Title/Abstract] OR "model*"[Title/Abstract] OR "score*"[Title/Abstract] OR "system*" OR "risk assessment"[Title/Abstract] OR "risk prediction"[Title/Abstract] OR "risk index"[Title/Abstract] OR "risk score"[Title/Abstract] OR "Predict*"[Title/Abstract] OR "risk calculation"[Title/Abstract] OR "machine learning"[Title/Abstract] OR "artificial intelligence"[Title/Abstract] OR "algorithm"[Title/Abstract] OR "deep learning"[Title/Abstract] OR "score" [Title/Abstract])) | 1199 |
| Web of Science (All databases, From database establishment to September 12, 2025) | #1  TS=("Hepatitis B Virus-Related hepatocellular carcinoma" OR "HBV-hepatocellular carcinoma" OR "HBV-HCC" OR "Hepatitis B virus-related HCC" OR "HBV positive HCC" OR "Hepatitis-B Virus-Associated Hepatocellular Carcinoma" OR "HBV-Related Hepatocellular Carcinoma" OR "HBV-associated hepatocellular carcinoma ")  #2  TS=("Prognosis" OR "prognostic factors" OR "prognostic model*" OR "Prognostic Risk Prediction" OR "predict model" OR "prediction model*" OR "nomogram*" OR "model*" OR "risk assessment" OR "risk prediction" OR "risk index" OR "risk score"  OR "Predict*" OR "risk calculation" OR "machine learning" OR "artificial intelligence" OR "algorithm" OR "deep learning" OR "score")  #1 AND #2 | 1414 |
| Embase (From database establishment to September 12, 2025) | (('hepatitis b virus-related hepatocellular carcinoma':ti,ab,kw OR 'hbv-hepatocellular carcinoma':ti,ab,kw OR 'hbv-hcc':ti,ab,kw OR 'hepatitis b virus-related hcc':ti,ab,kw OR 'hbv positive hcc':ti,ab,kw OR 'hepatitis-b virus-associated hepatocellular carcinoma':ti,ab,kw OR 'hbv-related hepatocellular carcinoma':ti,ab,kw OR 'hbv-associated hepatocellular carcinoma':ti,ab,kw)) AND (('prognosis':ti,ab,kw OR 'prognostic factors':ti,ab,kw OR 'prognostic model*':ti,ab,kw OR 'prognostic risk prediction':ti,ab,kw OR 'predict model':ti,ab,kw OR 'prediction model*':ti,ab,kw OR 'nomogram*':ti,ab,kw OR 'model*':ti,ab,kw OR 'score*':ti,ab,kw OR 'system*' OR 'risk assessment':ti,ab,kw OR 'risk prediction':ti,ab,kw OR 'risk index':ti,ab,kw OR 'risk score':ti,ab,kw OR 'predict*':ti,ab,kw OR 'risk calculation':ti,ab,kw OR 'machine learning':ti,ab,kw OR 'artificial intelligence':ti,ab,kw OR 'algorithm':ti,ab,kw OR 'deep learning':ti,ab,kw OR 'score':ti,ab,kw)) | 1695 |
| Scopus (From database establishment to September 12, 2025) | ( TITLE-ABS-KEY ( "Hepatitis B Virus-Related hepatocellular carcinoma" OR "HBV-hepatocellular carcinoma" OR "HBV-HCC" OR "Hepatitis B virus-related HCC" OR "HBV positive HCC" OR "Hepatitis-B Virus-Associated Hepatocellular Carcinoma" OR "HBV-Related Hepatocellular Carcinoma" OR "HBV-associated hepatocellular carcinoma" ) AND TITLE-ABS-KEY ( "Prognosis" OR "prognostic factors" OR "prognostic model*" OR "Prognostic Risk Prediction" OR "predict model" OR "prediction model*" OR "nomogram*" OR "model*" OR "risk assessment" OR "risk prediction" OR "risk index" OR "risk score" OR "Predict*" OR "risk calculation" OR "machine learning" OR "artificial intelligence" OR "algorithm" OR "deep learning" OR "score" )) | 1424 |
| Ovid MEDLINE(R) and Epub Ahead of Print, In-Process, In-Data-Review & Other Non-Indexed Citations, Daily and Versions 1946 to September 12, 2025 | ("Prognosis" or "prognostic factors" or "prognostic model?" or "Prognostic Risk Prediction" or "predict model" or "prediction model?" or "nomogram" or "model?" or "risk assessment" or "risk prediction" or "risk index" or "risk score" or "Predict?" or "risk calculation" or "machine learning" or "artificial intelligence" or "algorithm" or "deep learning" or "score").ab,bt,hw,kf,ot,sy,ti,fx,mx,nm,ox,px,rx,ui,ux. AND ("Hepatitis B Virus-Related hepatocellular carcinoma" or "HBV-hepatocellular carcinoma" or "HBV-HCC" or "Hepatitis B virus-related HCC" or "HBV positive HCC" or "Hepatitis-B Virus-Associated Hepatocellular Carcinoma" or "HBV-Related Hepatocellular Carcinoma" or "HBV-associated hepatocellular carcinoma").ab,bt,hw,kf,ot,sy,ti,fx,mx,nm,ox,px,rx,ui,ux. | 1071 |

Table S3: List of data items extracted for each included study

| - Study Authors - Title - Published year - Lead authors contact details - Country in which the study conducted - Study funding sources - Possible conflicts of interest for study authors - Aim of study - Study design - Enrollment period for the study population - Study setting - Study Inclusion criteria - Study Exclusion criteria - Modeling method - Total number of participants - Sample size of training cohort and validation cohort - Number of recurrence events - Number of candidate and final predictors - Method for selection of candidate predictors - Method for selection of final predictors during multivariable modelling (e.g., full model approach, backward or forward selection, stepwise) - Final predictors - Prediction outcome - Handing of missing data (imputation/complete case analysis/not mentioned) - Method used for test model performance (Overall performance, discrimination, calibration, reclassification, clinical usefulness) - Discrimination: C-index, Area under the curves (AUC) in receiver operating characteristic curves (ROC) - Calibration: Calibration curve, Calibration slope, Hosmer-Lemeshow test (H-L test) - Clinical usefulness: Net Benefit (NB), Decision curve analysis (DCA) - Model validation: Internal validation (random split-sample, bootstrap and cross-validation) or External validation (temporal validation, geographical validation) - Model presence (nomogram, scoring system, web calculator, mathematical formula) - Was there any report of implementation and change in clinical practice - Key conclusions of study authors: interpretation of presented models-useful for practice vs exploratory, comparison with other studies, strengths, and limitations - References to other relevant studies - Correspondence required for further study information |
| --- |

Table S4: Characteristic of included studies (inclusion and exclusion criteria)

| **Author, year** | **Inclusion criteria** | **Exclusion criteria** |
| --- | --- | --- |
| Ying Zhang 2025 | (1) Patients in whom HBV-HCC was clearly diagnosed; (2) Without previous treatment related to HC; (3) MRI was performed before treatment; (4) Absence of other tumors. HBV-HCC was diagnosed according to the 2018 Practice Guidance by the American Association for the Study of Liver Diseases (AASLD). | (1) Patients who had received other treatments, such as transcatheter arterial chemoembolization (TACE); (2) Patients with incomplete information; (3) Those that were lost to follow-up; and (4) Cases with poor MRI images. |
| Yu Zhu 2024 | (1) Pathologically diagnosed HCC; (2) Lack of tumor thrombi in hepatic vein, portal vein, bile duct, and inferior vena cava; (3) Lack of invasion of adjacent organs, hilar lymph nodes, and distant metastases; (4) Complete tumor tissue specimens; (5) Patients on whom only surgical treatment was performed, and no transcatheter arterial chemoembolization (TACE), radiofrequency, or microwave treatment was performed before or during operation. | (1) Simultaneous presence of other malignant tumors; (2) Perioperative death; (3) No history of hepatitis B or hepatitis C virus anti-body positivity; (4) Patients undergoing nonradical surgery; (5) Patients with incomplete pathological and clinical data; (6) Lack of follow up for survival. |
| Yiqi Xiong 2024(1) | (1) Patients with preoperative HBV-DNA level 20–100 IU/mL or <20 IU/mL; (2) Early liver cancer patients who achieved complete remission after TACE combined with ablation; (3) Patients with Child–Pugh A or B liver function; (4) All patients who had not received any other treatment before ablation. | (1) A diagnosis of other malignant diseases within the past five years; (2) Autoimmune liver disease; (3) Previous treatment with other drugs, such as Chinese patent medicine, immunomodulatory drugs, glucocorticoid therapy, or other immunosuppressive treatments; (4) Incomplete clinical follow-up data. |
| Yiqi Xiong 2024(2) | (1) Aged 18-80 years; (2) received TACE combined ablation; (3) Child-Pugh classification was class A or B; (4) All patients had not received any other therapeutics before ablation. | (1) With second primary malignant tumors; (2) Clinical follow-up data incomplete; (3) Advanced HCC. |
| Qi Wang 2024 | (1) Patients diagnosed with hepatitis B virus (HBV)-related primary HCC; (2) Received the combination of transcatheter arterial chemoembolization (TACE) and ablation treatment; (3) Complete clinical and follow-up data. | (1) Non-primary HCC; (2) Not HBV-related HCC; (3) Received other anti-tumor therapies before the combination treatment; (4) Suffering from other malignancies or systemic diseases; (5) Incomplete clinical or follow-up data. |
| Chongming Zheng 2023 | Not mentioned | (1) No radical surgical therapy during hospitalization; (2) Pathological findings of nonprimary hepatocellular carcinoma; (3) Combination with other malignant tumors; (4) Other antitumor treatment before surgery; (5) Non-HBV-associated HCC. |
| Shilei Bai 2023 | (1) Postoperative pathologically confirmed HCC with positive MVI; (2) Patients with Child score of grade A or B; (3) No preoperative antitumor therapy; (4) The surgical approach was radical LR (R0 resection); (5) No large vessel invasion. | (1) Incomplete clinical data; (2) Tumor recurrence within 1 month or patient death within 3 months after surgery; (3) Patient combined with other tumor history; (4) Bile duct tumor thrombosis. |
| Zehao Zheng 2023 | (1) Had undergone liver resection and pathologically diagnosed with HBV-HCC; (2) Had liver function by the Child‒Pugh A and American Society of Anaesthesiologists (ASA) Score < III; and (3) Had not received any neoadjuvant treatments before hepatectomy. | (1) Had incomplete baseline data and follow-up data; (2) Had a positive surgical margin; (3) Had distant metastasis or adjacent organ invasion; and (4) Had received neoadjuvant treatments, such as TACE and HAIC |
| Jiasi Zhang 2023 | (1) The pathological diagnosis was HCC only (i.e., not combined HCC or cholangiocarcinoma); (2) Hepatitis B surface antigen (HBsAg) positivity; (3) Complete clinicopathologic and follow-up data; and (4) Underwent curative hepatectomy for the first time and had not received any other treatments before the operation. | (1) Patients with recurrent HCC; (2) Patients with extrahepatic metastasis or macroscopic major portal or hepatic vein tumor thrombus; and (3) Recurrence occurred within 1 month after the hepatectomy. |
| Chao Wang 2022 | The patients had hepatitis B virus (HBV)-related HCC and underwent LT. | Patients without HCC cirrhosis, those who underwent LT because of concomitant hepatocellular cholangiocarcinoma, those who died in the perioperative period because of surgical complications, and those with other diseases were not included |
| Zili Hu 2022 | (1) Age 18 to 75 years; (2) Primary resectable HBV-related HCC; (3) Histological confirmation of HCC; (4) Liver function at Child-Pugh class A; (5) An Eastern Cooperative Oncology Group (ECOP) performance status of 0. | (1) Coinfection with hepatitis virus C; (2) Received preoperative treatment, including interventional therapy, radiofrequency ablation, etc.; (3) Metastasis to extrahepatic sites, including lymph nodes, lung, etc.; (4) Patients with incomplete clinical data; (5) Patients who were lost to follow-up within 3 months after hepatectomy; (6) History of other malignancies; (7) Non-R0 liver resection. |
| Jin Gu 2022 | Hepatitis B virus- (HBV-) related HCC and Child-Pugh grade A liver function who underwent curative hepatectomy. | Not mentioned |
| Wei Shuyao 2021 | (1) Hepatitis B antigen positive; (2) Diagnosis of A or B, based on the Child–Pugh staging system (score ≤ seven); (3) Performance of preoperative abdominal MRI or CT contrast enhanced scan; (4) Confirmation of HCC in postoperative pathology. | (1) Hepatitis C antigen positive; (2) Performance of preoperative antitumor therapy (transcatheter arterial chemoembolization (TACE), radiotherapy, chemotherapy, etc.); (3) History of malignant tumors other than HCC; (4) Incomplete clinical data. |
| Yujing Xin 2021 | (1) HCC diagnosed by histopathology or noninvasive diagnostic guidelines; (2) Performance status of 0 or 1 based on Eastern Cooperative Oncology Group score; (3) EHCC patients with chronic HBV infection (solitary tumor ≤3 cm or up to three tumors ≤3 cm); (4) Complete tumor ablation; (5) Positive hepatitis B surface antigen (HBsAg) for at least 6 months. | (1) Patients who received other curative treatment including hepatic resection or hepatic transplantation; (2) Patients with other types of hepatitis (e.g., alcoholic hepatitis, hepatitis A or C, nonalcoholic fatty liver disease, etc.); and (3) Patients who died or developed  HCC recurrence within 2 years after RFA were also excluded, as this study focused on late recurrence. |
| Mingyang Bao 2021 | (1) Patients undergoing liver resection for HCC diagnosed pathologically; (2) HBV surface antigen (HBsAg)- and/or HBV core antibody (HBcAb)-positive patients; (3) Child–Pugh class A or B (score ≤7) patients; (4) Patients performing preoperative abdominal contrast enhanced magnetic resonance imaging (MRI) and/or contrast enhanced computed tomography (CT) scan; (5) Anatomical and non-anatomical hepatectomies before July 1, 2018. | (1) Patients undergoing more than one additional procedure in the liver; (2) Patients with a history of tumors; (3) HCV-positive patients; (4) Patients with incomplete clinical data; (5) Patients receiving antitumor therapy before operation [i.e., transarterial chemoembolization (TACE), radiotherapy, or chemotherapy]. |
| Xiangkun Wang 2019 | Not mentioned | Not mentioned |
| Jong Man Kim 2019 | (1) Histologically confirmed HBV-related HCC; (2) Performance status score of 0 to 1; (3) No evidence of extrahepatic metastasis; (4) No history of other malignancies; (5) Curative resection; and (6) HCC based on preoperative radiologic images. | (1) Etiology other than HBV; (2) Mixed HCC and cholangiocarcinoma on pathology; (3) Age <18years; (4) Palliative hepatic resection; (5) Concurrently intraoperative RFA during surgical resection; (6) Fibrolamellar HCC; (7) Death caused by severe surgical complications; (8) Missing or incomplete data; (9) History of preoperative locoregional therapies such as liver resection, transarterial chemoembolization, radiofrequency ablation, or radiation; or (10) Loss to follow-up after hepatectomy. |
| Lingling He 2019 | (1) HBV-related HCC; (2) A single isolated small (3cm in diameter) primary tumor; (3) 18–75years of age; and (4) Data were available for at least 1year of follow-up after surgery. | (1) Evidence of hepatitis C virus (HCV) or human immunodeficiency virus (HIV) infection; (2) Severe disease or dysfunction of the heart, lungs, brain, kidneys or other vital organs; (3) Severe mental illness; (4) Pregnancy/lactation; (5) Incomplete clinical data. |
| Abdulahad Abdulrab Mohammed Al-Ameri 2019 | (1) Adult patients with age≥18 (2) Preoperative radiologically diagnosed HCC depending on guidelines of the current guidelines of American Association for the Study of Liver Diseases (AASLD) (3) No history of previous LT or combined hepatorenal transplantation; (4) Patients who survived at least 3 months after the date of surgery; (5) No incidental HCC; (6) All the clinical and laboratory data required for the analysis are available. | Not mentioned |
| Wei Qin 2018 | All HBV-related HCC patients were underwent R0 resection and not received pre-operative treatment (ie. liver transplant, transarterial chemoembolization, or radiofrequency ablation) | Not mentioned |
| Rui Liao 2018 | (1) All patients tested HBV surface antigen (HBsAg) and HBV-DNA loads; (2) Reliable laboratory test data including liver function; (3) The absence of preoperative extrahepatic metastases confirmed by computed tomography (CT) and/or magnetic resonance imaging (MRI) scanning; (4) No preoperative anticancer therapies; (5) Complete resection of all tumor nodules; (6) Complete patient records and follow-up data; (7) Survival for more than 30 days after surgery. | Patients were excluded if they had any infection and autoimmune disease or anticancer therapies before operation. |
| Ivan Fan-Ngai Hung 2016 | HBV-related HCC who underwent curative tumor resection patients were either at stage 0 or A, according to the Barcelona Clinic Liver Cancer Staging upon resection. | Patients with other concomitant liver diseases including hepatitis C or D viral infection, autoimmune hepatitis, Wilson’s disease, primary biliary cirrhosis, alcoholic liver disease, fatty liver (diagnosed by ultrasonography) and extrahepatic metastasis diagnosed before resection were excluded from the study. |

Table S5a: Basic information of 22 included models (Author, published year, modelling methods, sample size of training and validation cohort, number of candidate and final predictors, selection of candidate and final predictors)

| **Author, year** | **Modeling methods** | **Sample size** | | **No. predictors** | | **Selection of candidate predictors** | **Selection of final predictors** |
| --- | --- | --- | --- | --- | --- | --- | --- |
|  |  | Training | Validation | Cand. | Final. |  |  |
| Ying Zhang 2025 | Logistic regression | 116 | 50 | 15 | 3 | Univariate logistic regression | Full model approach |
| Yu Zhu 2024 | Cox regression | 465 | 215 | 31 | 3 | Univariate cox regression | Stepwise selection |
| Yiqi Xiong 2024(1) | Cox regression | 282 | 121 | 31 | 4 | Lasso Regression | Full model approach |
| Yiqi Xiong 2024(2) | Cox regression | 385 | 289 | 8 | 6 | Lasso Regression | Full model approach |
| Qi Wang 2024 | Machine learning | 172 | 75 | 6 | 3 | XGBoost, RSF | Full model approach |
| Chongming Zheng 2023 | Cox regression | 86 | 38 | 30 | 4 | Univariate cox regression | Forward stepwise selection |
| Shilei Bai 2023 | Cox regression | 530 | 516 | 15 | 7 | Univariate Cox regression | Stepwise selection |
| Zehao Zheng 2023 | Cox regression | 349 | 234 | 17 | 4 | Univariate Cox regression | Full model approach |
| Jiasi Zhang 2023 | Cox regression | 393 | 210 | 18 | 5 | Univariate Cox regression | back selection |
| Chao Wang 2022 | Cox regression | 270 | 295 | 30 | 5 | Univariate Cox regression | Full model approach |
| Zili Hu 2022 | Cox regression | 616 | 308 | 26 | 5 | Univariate Cox regression | Full model approach |
| Jin Gu 2022 | Cox regression | 636 | | 10 | 5 | Univariate Cox regression | Full model approach |
| Wei Shuyao 2021 | Cox regression | 470 | 204 | 10 | 5 | Univariate Cox regression | Full model approach |
| Yujing Xin 2021 | Cox regression | 302 | 143 | 13 | 3 | Univariate Cox regression | Full model approach |
| Mingyang Bao 2021 | Cox regression | 675 | 252 | 24 | 8 | Univariate Cox regression | Two-way stepwise selection |
| Xiangkun Wang 2019 | Cox regression | 212 | | 9 | 4 | Univariate Cox regression | Full model approach |
| Jong Man Kim 2019 | Cox regression | 420 | | 15 | 8 | Univariate Cox regression | Stepwise regression |
| Lingling He 2019 | Cox regression | 203 | 64 | 5 | 4 | Univariate Cox regression | Full model approach |
| Abdulahad Abdulrab Mohammed Al-Ameri 2019 | Cox regression | 486 | 262 | 20 | 3 | Univariate Cox regression | Backward selection |
| Wei Qin 2018 | Cox regression | 162 | 176 | 21 | 3 | Univariate Cox regression | Full model approach |
| Rui Liao 2018 | Cox regression | 342 | 310 | 9 | 6 | Univariate Cox regression | Backward step-down selection |
| Ivan Fan-Ngai Hung 2016 | Cox regression | 200 | | 11 | 5 | Univariate Cox regression | Stepwise selection |

XGBoost: EXtreme Gradient Boosting, RSF: Random Survival Forest.

Table S5b: Basic information of 22 included models (final predictors, prediction outcome, handing of missing data, model validation, model performance, model presence)

| **Author, year** | **Final predictors** | **Prediction Outcome** | **Handling of missing data** | **Model validation** | **Model performance** | **Model Presence** |
| --- | --- | --- | --- | --- | --- | --- |
| Ying Zhang 2025 | Aspartate aminotransferase, Portal hypertension, Deep learning-based, Radiomics score | 1-year recurrence rate | Complete case analysis | Geographical Validation | AUC, Calibration curve, H-L test, DCA | Nomogram |
| Yu Zhu 2024 | Tumor diameters, Microvascular invasion, Alburnin level | 6, 12, 24-months RFS | Complete case analysis | Bootstrap | AUC, Calibration curve | Nomogram |
| Yiqi Xiong 2024(1) | Gender, The Barcelona Clinical Liver Cancer stage, Globulin, Monocyte-to-lymphocyte ratio | 1-, 3-, 5-year RFS | Complete case analysis | Split-sample Validation | C-index, Calibration curve, DCA | Nomogram |
| Yiqi Xiong 2024(2) | Age, the Barcelona Clinical Liver Cancer stage, Tumor size, Globulin, Gamma glutamyl transferase, Bileacids | 1-, 3-, 5-year RFS | Complete case analysis | Split-sample Validation and Geographical validation | C-index, AUC, Calibration curve, DCA | Nomogram |
| Qi Wang 2024^24^ | Age, Aspartate aminotransferase, Prothrombin time activity | 1-, 3-, 5-year RFS | Complete case analysis | Split-sample Validation | C-index, AUC Calibration curve, DCA | Nomogram |
| Chongming Zheng 2023 | TNM stage, Aspartate aminotransferase, Veillonella, Streptococcus pneumoniae | 1-year recurrence rate | Complete case analysis | Temporal validation | C-index, AUC, Calibration curve, DCA | Nomogram |
| Shilei Bai 2023 | Neutrophil‐to‐lymphocyte ratio, HBV-DNA, Alpha-fetoprotein, Narrow surgical margins, Tumor diameter, Multiple tumors, Absence of postoperative TACE | 1-, 3-, 5-year recurrence rate | Complete case analysis | Split-sample Validation and Geographical validation | C-index, AUC, Calibration curve | Nomogram and Web-calculator |
| Zehao Zheng 2023 | Microvascular invasion, CNLC stage, Systemic inflammation response index, Aspartate aminotransferase to neutrophil ratio index | 1-, 2-, 3-year RFS | Complete case analysis | Geographical Validation | C-index, AUC, Calibration curve, DCA | Nomogram |
| Jiasi Zhang 2023 | Alpha-fetoprotein, HBV-DNA, Satellite nodules, Microvascular invasion, Tumor grade | 2-, 3-, and 5-year RFS | Complete case analysis | Split-sample Validation | C-index, Calibration curve, DCA | Nomogram |
| Chao Wang 2022 | Milan criteria, Alpha-fetoprotein, Microvascular invasion, D.dimer, Fibrinogen | 1-, 3-year rate | Complete case analysis | Geographical Validation | AUC | Nomogram |
| Zili Hu 2022 | Tumor size, Tumor number, Microvascular invasion, Tumor differentiation, Lactate dehydrogenase | 1-, 3-, 5-year rate | Complete case analysis | Split-sample Validation and Geographical validation | C-index, AUC, Calibration curve | Nomogram |
| Jin Gu 2022 | Cirrhotic severity scoring, Alpha-fetoprotein level, Tumor number, Tumor size, Macrovascular invasion | 3-, 5-year RFS | Not mentioned | Bootstrap | C-index, Calibration curve | Nomogram |
| Wei Shuyao 2021 | Cytokeratin 19, postoperative platelets, Satellite nodules, Microvascular invasion, Tumor boundary (complete/incomplete) | 2-year RFS | Complete case analysis | Split-sample Validation and Geographical validation | C-index, Calibration curve | Nomogram |
| Yujing Xin 2021 | Age-male-ALBI platelets score, Tumor number, Preoperative HBV-DNA level | 3-, 4-, and 5-year RFS | Not mentioned | Bootstrap and Geographical validation | C-index, AUC, Calibration curve | Nomogram |
| Mingyang Bao 2021 | HBV DNA, Arterial phase, surrounding satellite nodules, Microvascular invasion, Preoperative total protein, Preoperative direct bilirubin, Postoperative platelets, and Postoperative alkaline phosphatase | 1, 2-year recurrence rate | Complete case analysis | Bootstrap and Geographical validation | C-index, AUC, Calibration curve, H-L test, DCA | Nomogram |
| Xiangkun Wang 2019 | Gender, Cirrhosis, the Barcelona Clinical Liver Cancer stage, Phospholipase C β1 | 1-, 3-, 5-year RFS | Not mentioned | External database validation | AUC | Nomogram |
| Jong Man Kim 2019 | Free resection margin, Tumor number, PIVKA-II, Albumin, Portal vein tumor thrombosis, Intrahepatic Meta, Alanine phosphatase, Hemorrhage | 1-, 3-, 5-year RFS | Complete case analysis | Bootstrap | C-index, Calibration curve | Nomogram |
| Lingling He 2019 | History of alcoholism, γ-glutamyl transpeptidase, Total protein, Alpha fetoprotein | 1-year recurrence rate | Complete case analysis | Temporal Validation | AUC | Risk score formula |
| Abdulahad Abdulrab Mohammed Al-Ameri 2019 | Pre-LT alpha fetoprotein, the largest diameter of tumor, number of nodules (single vs multiple) | 2-year recurrence rate | Complete case analysis | Split-sample Validation | C-index | Score system |
| Wei Qin 2018 | Fibrosis-4, Total tumor volume, and differentiation grade (I/II/III/IV) | 1-, 3-, 5-year RFS | Not mentioned | Split-sample Validation and Geographical validation | AUC | Score system |
| Rui Liao 2018 | Alpha-fetoprotein, Tumor number, Tumor size, Microvascular invasion, HBV-ALBI, HBV-PIS | 1-, 3-, 5-year RFS | Complete case analysis | Split-sample Validation and Geographical validation | C-index, AUC, Calibration curve | Nomogram |
| Ivan Fan-Ngai Hung 2016 | HBV-DNA, The presence of lymphovascular permeation, The presence of microsatellite lesions, Alpha-fetoprotein before resection | 1-, 3-year recurrence rate | Not mentioned | Cross-Validation | AUC | Score system |

RFS: Recurrence-free survival, TACE: Transcatheter arterial chemoembolization, CNLC: China liver cancer staging, TNM: Tumor Node Metastasis

Table S6: Subgroup analysis pooled C-indexes

| **Category** | **Studies** | **C-index (95% CI)** | ***P*** |
| --- | --- | --- | --- |
| **Published year** |  |  | 0.63 |
| After 2022 | 11 | 0.722 (0.689-0.753) |  |
| Before2022 | 6 | 0.736 (0.688-0.779) |  |
| **Prediction outcome** |  |  | **0.02** |
| Recurrence rate | 6 | 0.757 (0.729-0.783) |  |
| RFS | 11 | 0.707 (0.673-0.739) |  |
| **Number of predictors** |  |  | 0.54 |
| ≥5 | 12 | 0.733 (0.708-0.758) |  |
| <5 | 5 | 0.719 (0.631-0.778) |  |
| **Model validation type** |  |  | 0.35 |
| Internal validation | 10 | 0.716 (0.680-0.750) |  |
| External validation | 7 | 0.741 (0.701-0.777) |  |

RFS: Recurrence-Free Survival

Table S6: Abbreviations

| HBV-HCC | Hepatitis B virus-associated Hepatocellular Carcinoma |
| --- | --- |
| HCC | Hepatocellular Carcinoma |
| HBV | Hepatitis B Virus |
| PRISMA | Preferred Reporting Items for Systematic Reviews and Meta-Analyses |
| CHARMS | Checklist for Critical Appraisal and Data Extraction for Systematic Reviews of Prediction Modelling Studies |
| PROBAST | Prediction Model Risk of Bias Assessment Tool |
| t-AUC | Time-dependent Area Under the Curve |
| ROC | Receiver Operating Characteristic Curves |
| C-index | Concordance Index |
| CI | Confidence Interval |
| ML | Machine Learning |
| RFS | Recurrence-Free Survival |
| XGBoost | EXtreme Gradient Boosting |
| RSF | Random Survival Forest |
| H-L | Hosmer-Lemeshow |
| MVI | Microvascular Invasion |
| AFP | Alpha-fetoprotein |
| BCLC | Barcelona Clinic Liver Cancer |
| EPV | Events Per Variable |
| DCA | Decision Curve Analysis |
| TRIPOD | Transparent Reporting of a Multivariable Prediction Model for Individual Prognosis or Diagnosis |

Figure S1: Forest plot of subgroup analysis


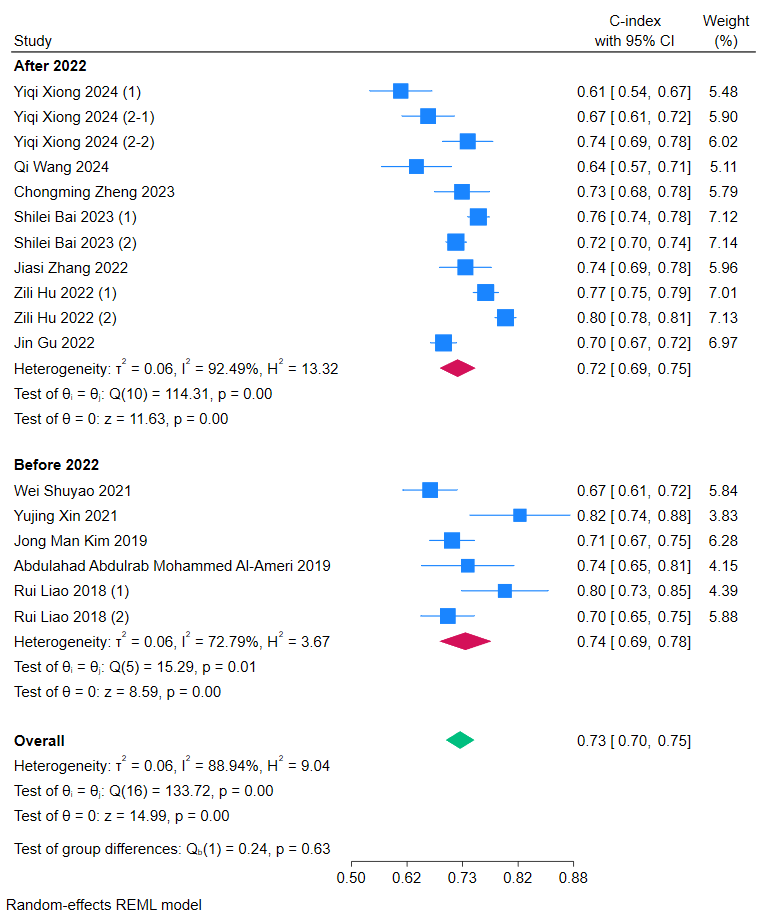


(a) Published year


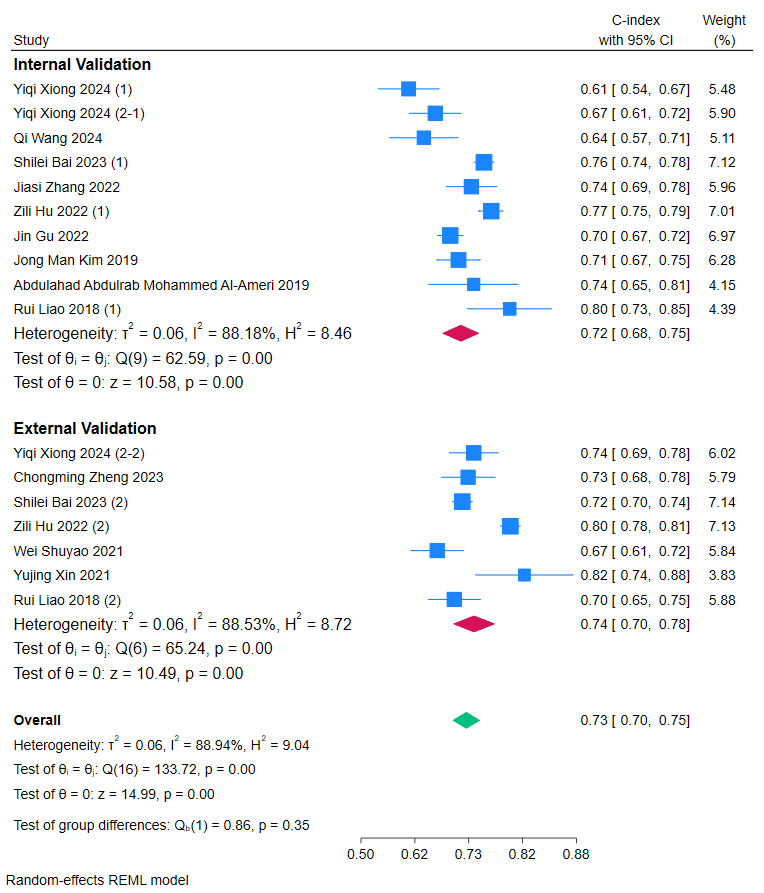


(b) Validation type


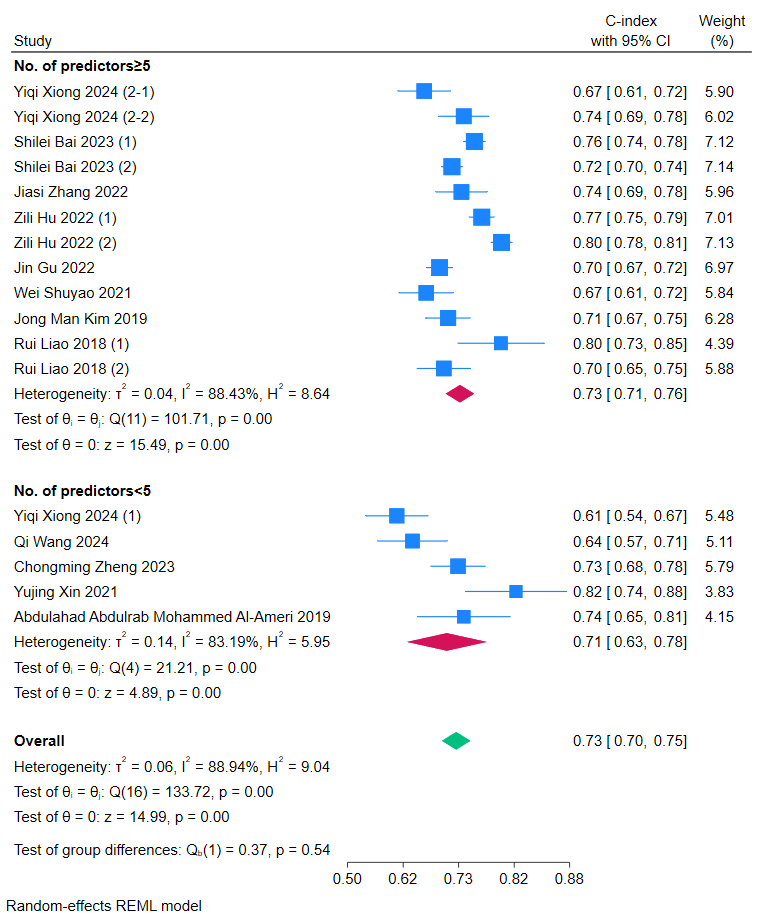


(c) Number of predictors


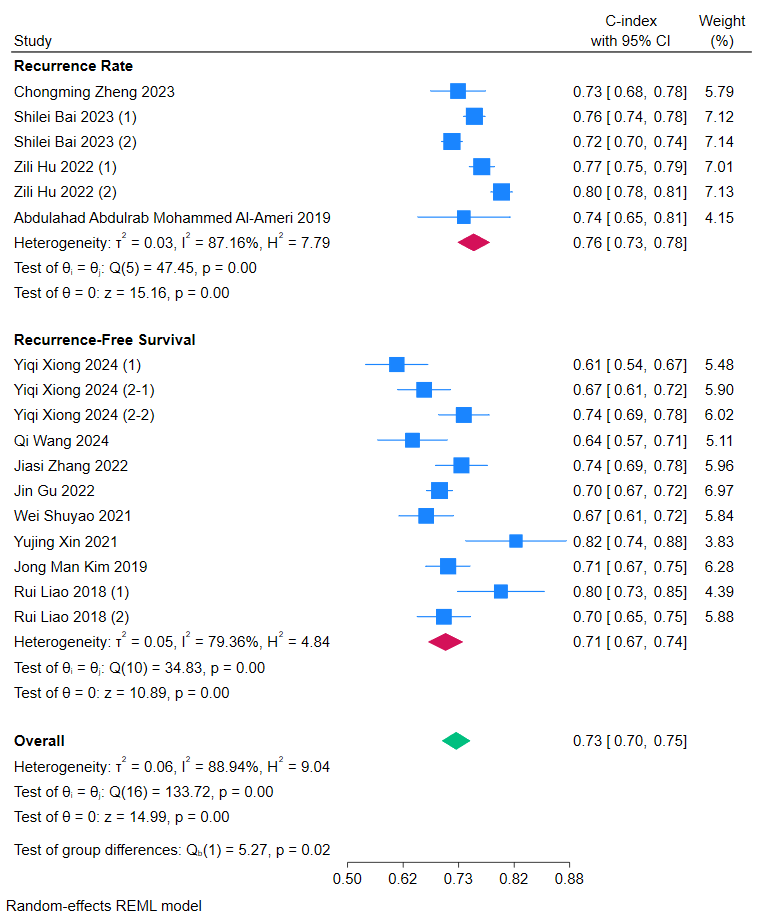


(d) Prediction outcome

Figure S2: Meta-regression analysis exploring for heterogeneity

| 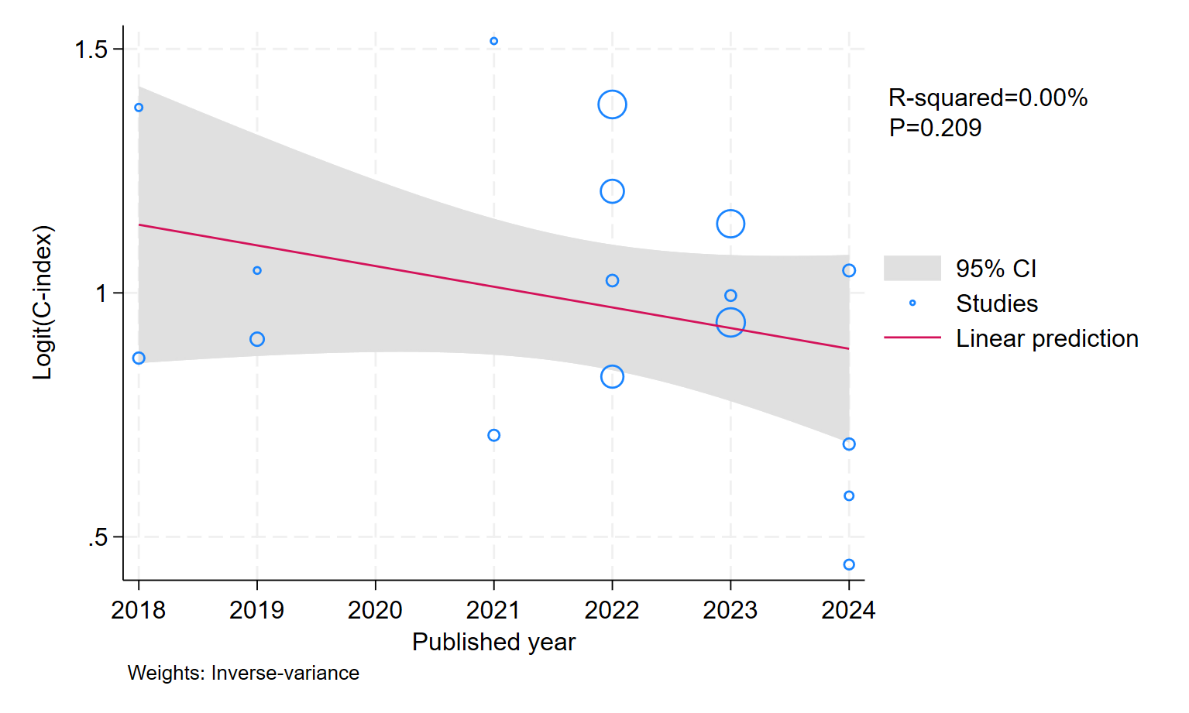  (a) Published year |
| --- |
| 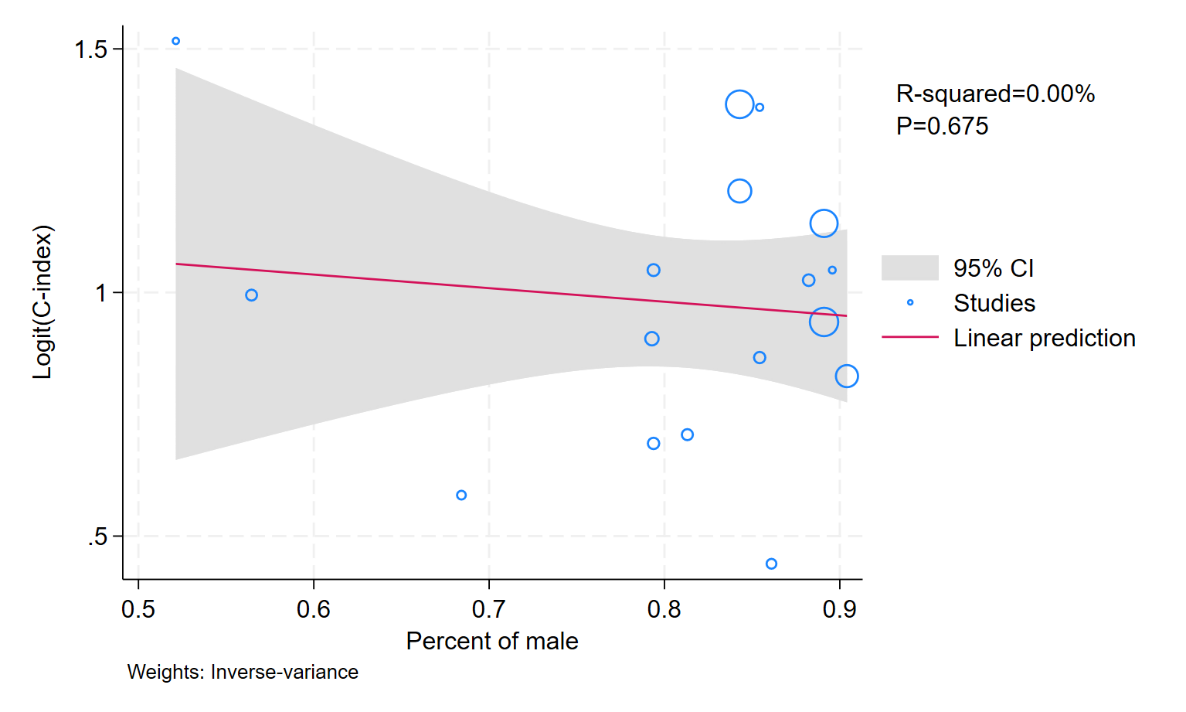  (b) Percent of male |
| 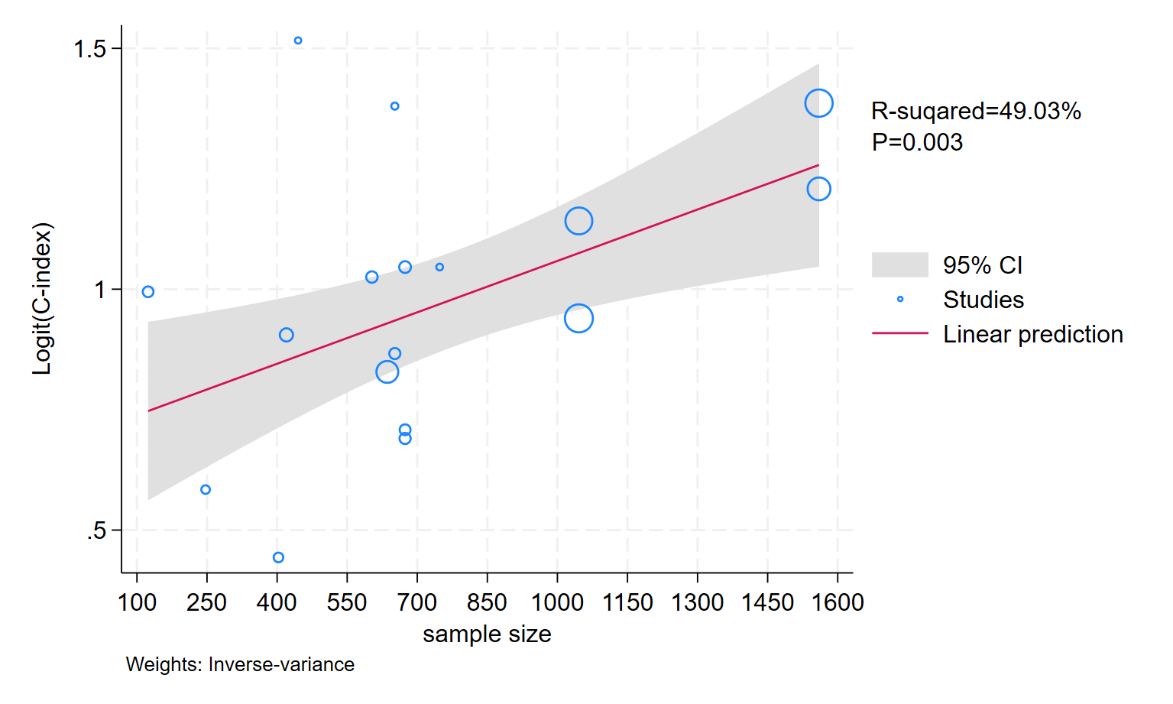  (c) Model sample size |
| 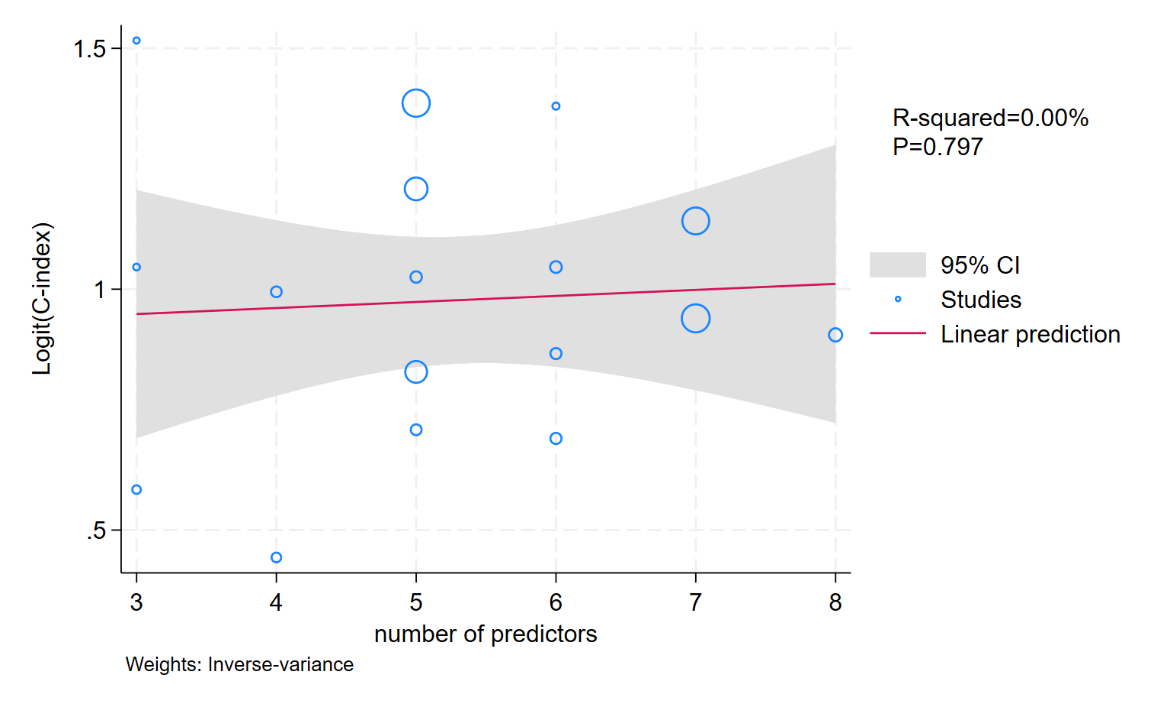  (d) Number of predictors |

Figure S3: Sensitivity analysis of pooled C-indexes


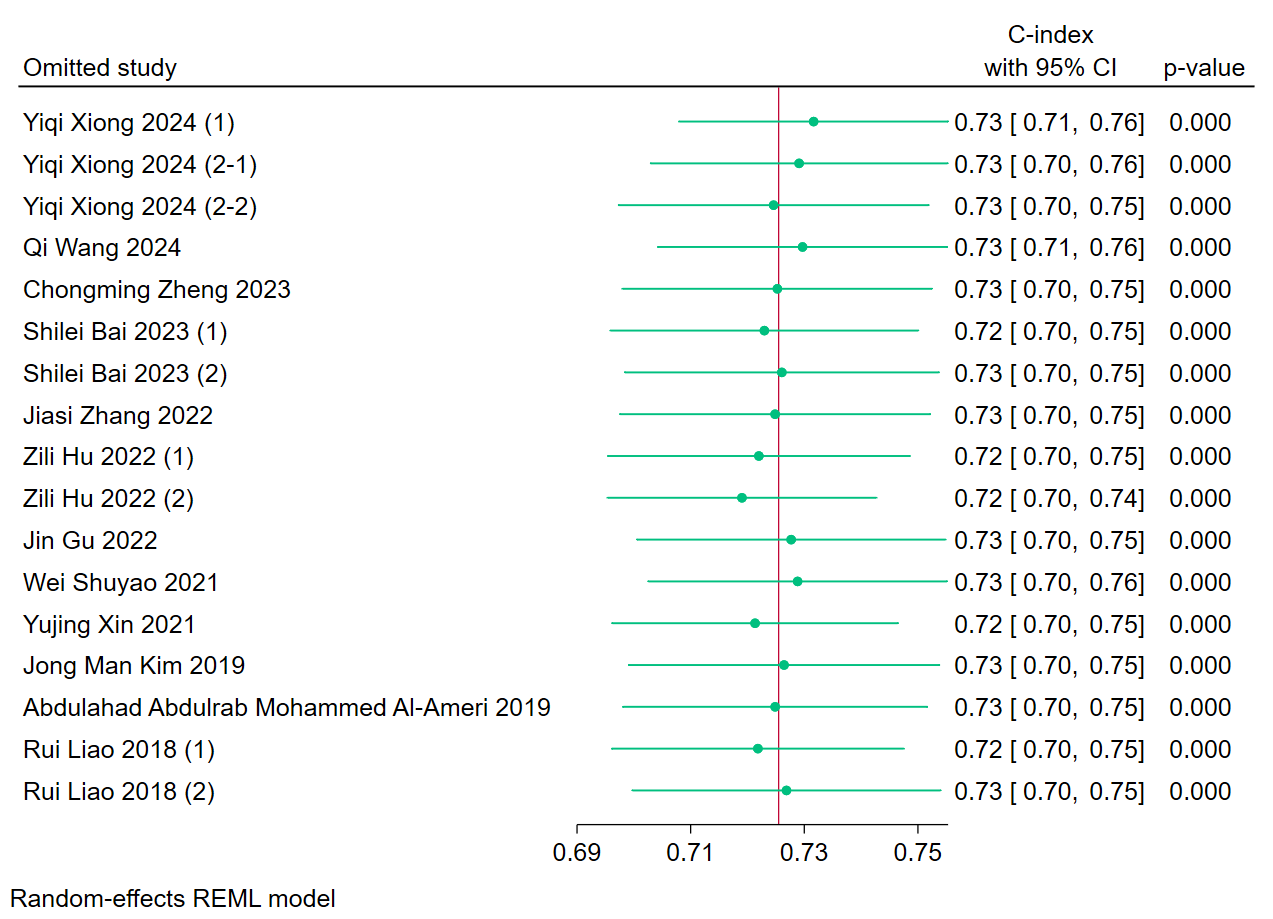


Figure S4: Publication bias results for Egger’s test


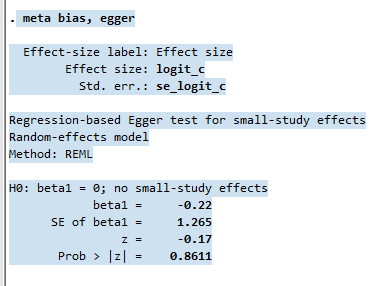


Figure S5: Funnel plot


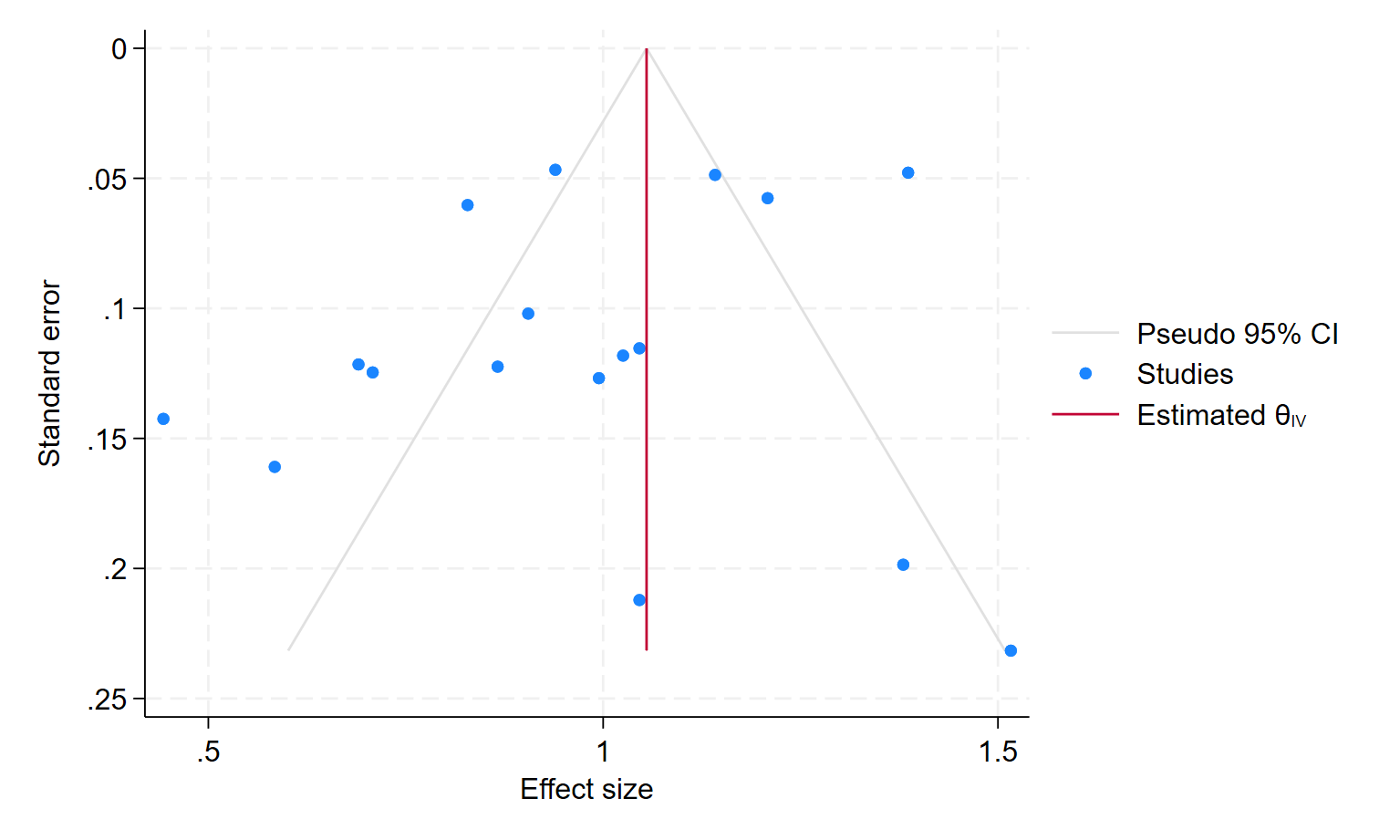


Figure S6 Risk of bias of four domains


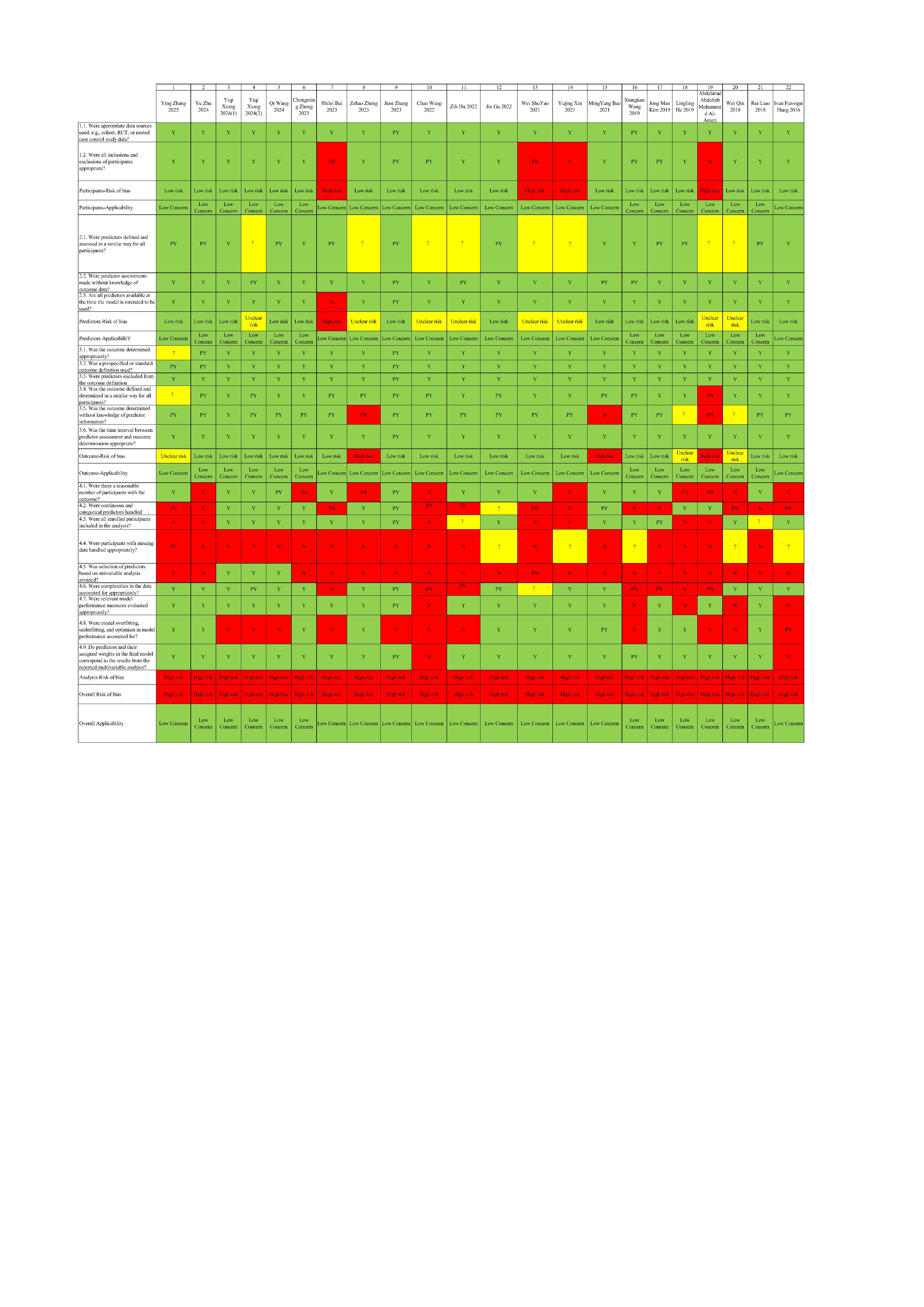


Y=Yes, N=No, PY=Probably yes, PN=Probably no, “?”=Unclear
